# Hygroscopic properties of particulate matter and effects of their interactions with weather on visibility

**DOI:** 10.1038/s41598-021-95834-6

**Published:** 2021-08-12

**Authors:** Wan-Sik Won, Rosy Oh, Woojoo Lee, Sungkwan Ku, Pei-Chen Su, Yong-Jin Yoon

**Affiliations:** 1 School of Mechanical and Aerospace Engineering, Nanyang Technological University, Singapore, 639798 Singapore; 2 Department of Industrial and Systems Engineering, Korea Advanced Institute of Science and Technology (KAIST), Daejeon, 34141 Korea; 3 Department of Public Health Sciences, Graduate School of Public Health, Seoul National University, Seoul, 08826 Korea; 4 Department of Aviation Industrial and System Engineering, Hanseo University, Seosan-si, Chungcheongnam-do 32158 Korea; 5 Department of Mechanical Engineering, Korea Advanced Institute of Science and Technology (KAIST), Daejeon, 34141 Korea

**Keywords:** Environmental sciences, Engineering

## Abstract

The hygroscopic property of particulate matter (PM) influencing light scattering and absorption is vital for determining visibility and accurate sensing of PM using a low-cost sensor. In this study, we examined the hygroscopic properties of coarse PM (CPM) and fine PM (FPM; PM_2.5_) and the effects of their interactions with weather factors on visibility. A censored regression model was built to investigate the relationships between CPM and PM_2.5_ concentrations and weather observations. Based on the observed and modeled visibility, we computed the optical hygroscopic growth factor, $f\left( {RH} \right)$, and the hygroscopic mass growth, $GM_{VIS}$, which were applied to PM_2.5_ field measurement using a low-cost PM sensor in two different regions. The results revealed that the CPM and PM_2.5_ concentrations negatively affect visibility according to the weather type, with substantial modulation of the interaction between the relative humidity (RH) and PM_2.5_. The modeled $f\left( {RH} \right)$ agreed well with the observed $f\left( {RH} \right)$ in the RH range of the haze and mist. Finally, the RH-adjusted PM_2.5_ concentrations based on the visibility-derived hygroscopic mass growth showed the accuracy of the low-cost PM sensor improved. These findings demonstrate that in addition to visibility prediction, relationships between PMs and meteorological variables influence light scattering PM sensing.

Visibility represents the maximum distance from which an object can be recognized. It is controlled by the intensity of light absorption and scattering of gases and particulates in the atmosphere. Visibility degradation is mainly influenced by the amount and type of water droplets and aerosols suspended in the air^1,2^. This degradation affects people psychologically and physiologically, and causes inconvenience and poses danger when performing human activities such as aviation and shipping^1,3^. Sudden and prolonged visibility impairment poses risks to traffic areas, particularly aviation safety, by restricting surface movements and flight conditions during a flight’s takeoff or landing at airports^4,5^. In recent decades, due to increasing particulate matter (PM) concentrations from air pollutants in industrial areas and automobile exhaust in urban areas, visibility impairment, characterized by heavy haze or a haze-fog mixture, has increased continually^6,7^. These anthropogenic emissions interacting with meteorological variables complicate visibility prediction.

PM is mainly classified by size as PM_10_ and PM_2.5_, characterized by aerodynamic diameters of less than 10 and 2.5 µm, respectively. According to its definition, PM_2.5_ is a subset of PM_10_ particles. Thus, PM_10–2.5_, which has a particulate diameter between 2.5 and 10 µm, is termed as coarse PM (CPM) as opposed to the fine PM (FPM; PM_2.5_). The CPM originates primarily from nature (such as the ocean and land), whereas PM_2.5_ are generally secondary products of air pollution, in addition to the natural sources^8–10^. Prior to PM_2.5_ attracting significant attention because of its effects on human health and environment, PM_10_ was the most commonly monitored pollutant and was employed to study the pollution trends over past decades or to predict visibility^11–14^. Currently, the mitigation and monitoring of PM_2.5_ is the primary concern. Consequently, visibility studies also focus on the chemical composition and properties of PM_2.5_^15–17^.

Owing to its abundant water-soluble particles, PM_2.5_ exhibits better hygroscopic properties for light scattering, thereby affecting visibility more than the CPM^14,17,18^. Water vapor impacts visibility because of the hygroscopic growth at elevated relative humidity (RH). The hygroscopic growth is the change of the diameter of a particle because of the uptake of water. It is represented as the particle size hygroscopic growth factor, $GF\left( {RH} \right) = D\left( {RH} \right)/D_{d}$, defined as the ratio of the diameter of a particle ($D$) at a given RH to that of dry particle ($D_{d}$)^10,19,20^. Similarly, the optical hygroscopic growth factor, $f\left( {RH} \right) = \sigma_{ext} \left( {RH} \right)/\sigma_{ext} \left( {dry} \right)$, is defined as the ratio of the extinction coefficient ($\sigma_{ext}$) under wet conditions to that under dry conditions^21–23^. Hygroscopic PM scatters more light under humid weather conditions, resulting in lower visibility at high RH levels; therefore, predicting visibility under PM effects while considering their complicated interaction with meteorological factors is still challenging^13,24^. It is already well documented that visibility is predicted primarily by RH and the chemical composition of aerosols with empirical functions^21,22^. While chemical species are not typically monitored outside of special campaign periods, PM and weather variables such as RH are regularly reported by environmental or weather authorities. An international airport is a suitable weather station for regularly conducting meteorological observations like visibility^25^. Thus, it is notable that visibility can be estimated in real-time by an empirical function from RH and PM concentration than by chemical species data. Meanwhile, the light scattering characteristics of PM are also valuable not only for visibility studies but for manufacturing PM measuring instruments^26–28^. Recently, low-cost PM sensors have become more popular due to the convenience of real-time air quality monitoring^29–31^. Although they are easy to operate, the low-cost PM sensors have low accuracy because the estimations of PM concentrations made by the light-scattering sensors are affected by a variety of environmental parameters^26,28,32,33^. An important reason for the lower accuracies is that low-cost sensors lack the RH and temperature control functions; therefore, light scattering sensors overestimate the PM concentration affected by humidity^26,34^. Therefore, while understanding the hygroscopic properties of the CPM and PM_2.5_ and their relationship with weather is essential for predicting visibility, it can also contribute to enhancing sensor technology for more accurate measurements of PM concentrations.

In this study, the hygroscopic properties of CPM and PM_2.5_, and the effects on visibility due to their interactions with meteorological factors were investigated by modeling the relationship between CPM and PM_2.5_ concentration, and weather observations collected over four years from Incheon International Airport (ICN). A censored regression model was built to quantitatively predict the impact of CPM and PM_2.5_ on visibility under different meteorological conditions. We focus on determining the characteristics of CPM and PM_2.5_ that interact with meteorological factors and quantifying the relationship between PM concentration and visibility. Furthermore, we conducted PM_2.5_ field measurements in two different regions, namely Jeju, Korea and Singapore, to assess the effect of the RH on PM measurement by applying the RH-adjusted correction to the low-cost PM_2.5_ sensors based on the visibility-derived hygroscopic mass growth.

## Results

### PM–visibility weather dependence

**Figure 1:**
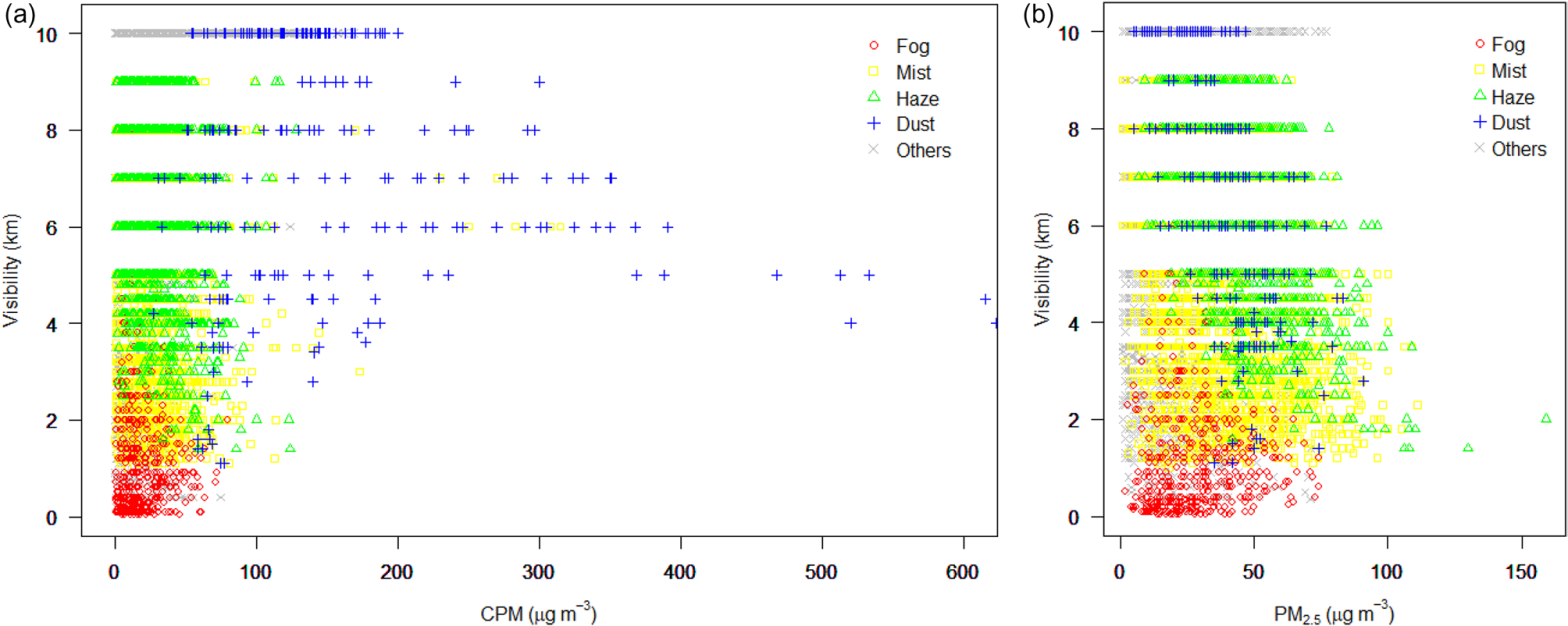
Scatter plots of visibility (km) and PM concentration (μg m^−3^) for CPM and PM_2.5_ at the ICN, 2015–2018. The different colors represent different weather types: haze, dust, mist, fog, and others (RA, DZ, SN, and None).

The distributions of PM concentrations and visibility data for 4 years at the ICN are displayed in Fig. 1. The visibility data represent hourly observations at the airport, while the PM concentrations were obtained from the Unseo air pollution monitoring station near the ICN. The highest visibility of 10 km implies equal to or more than 10 km, according to the visibility standards reported at airports^5,35^. The exceptionally high CPM concentrations in Fig. 1, which indicate the Asian Dust in February 2018, highlight the elevated CPM transportation into the Seoul Metropolitan Area (SMA). The data in Table S-1 reveal average PM_2.5_ concentration at the ICN of 23 μg m^−3^ over the four years. This value largely exceeds that of the WHO annual standard and those put forth by other countries^36,37^ (statistics for each variable are presented in Table S-1). The ICN is impacted by visibility impairment associated with high PM concentrations annually, and particularly from December 24–25, 2017, during which half of the flights were delayed or canceled due to heavy haze and dense fog with high PM concentrations^38^.

In Fig. 1, the plot of the relationships between the PM concentration and visibility under different weather types including haze (HZ), dust (DU), mist (BR), fog (FG), and others (RA (rain), DZ (drizzle), SN (snow), None) are represented by different colors (the criteria of each weather phenomena are presented in Table S-4). In the CPM plot, the boundary between dust and haze is distinct, whereas the PM_2.5_ plot exhibits an overlap. Although distinguishing between haze and mist in the CPM plot is difficult, the haze and mist distributions are easier to distinguish in the PM_2.5_ plot, and visibility in haze is higher than in mist for the PM_2.5_ plot. Consequently, the CPM concentration is remarkably high in dust, while haze is elevated in PM_2.5_. According to the weather type-based plot, visibility generally decreases with the PM concentration, except in the case of fog. Mist is at the boundary between haze and fog, and many cases in fog are characterized by low visibility, regardless of the PM concentration. The cases of fog exceeding 1 km involve scattered fog such as the *fog patches* and *partial fog,* for which the weather codes are BCFG and PRFG, respectively, according to the weather reporting manual^39^. The PM–visibility relationships under different meteorological conditions are further examined in the next section.

### Correlation between PM and meteorological factors

**Figure 2:**
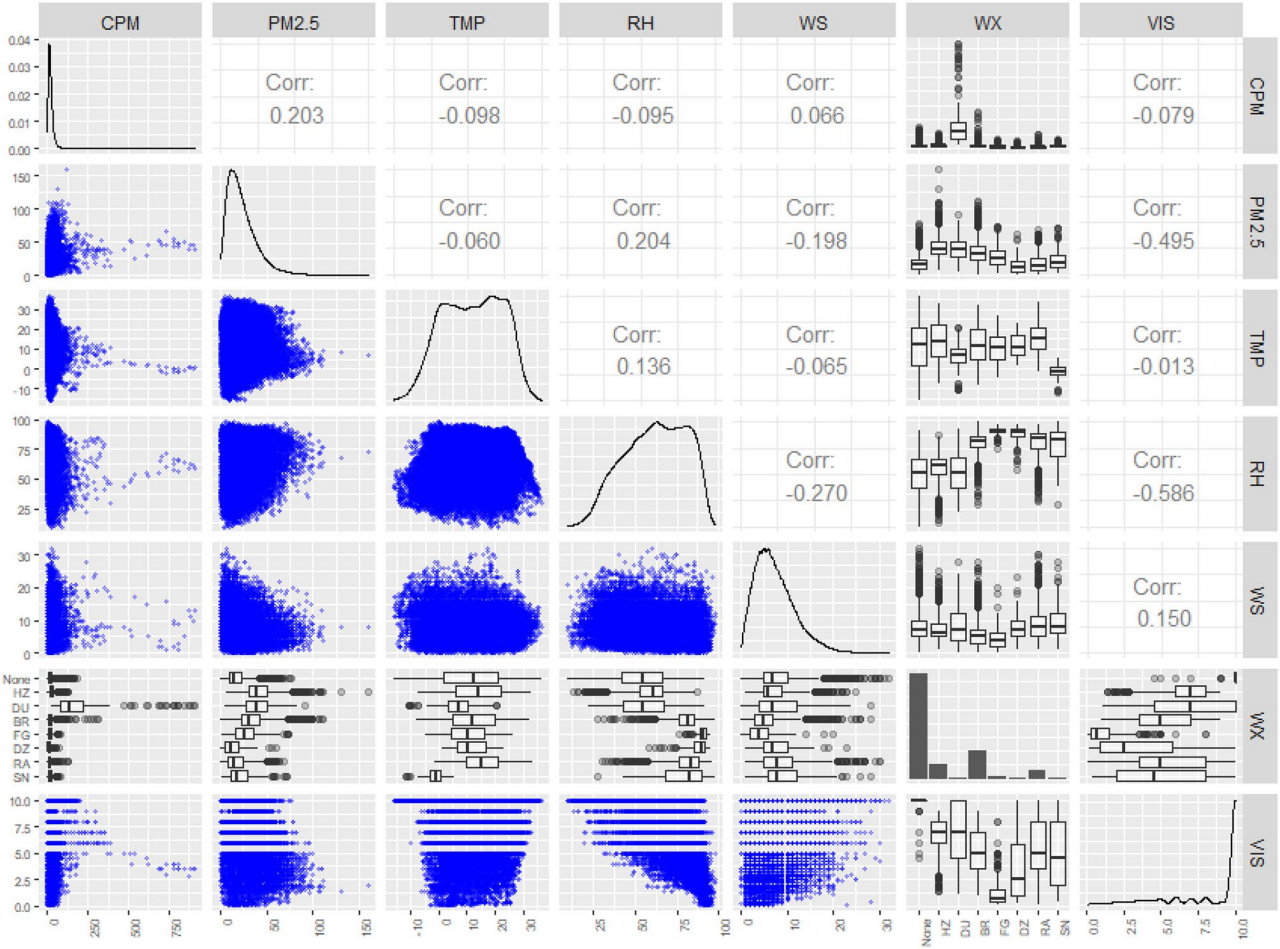
Matrix of plots and correlation coefficients between variables for the ICN over four years (2015–2018). The upper panel above the diagonal in each matrix shows correlation coefficients between two variables; the lower panel below the diagonal gives their scatter plots. The histograms of each variable are shown in the diagonal line. For WX (weather), the upper and lower panels (the two are the same) give boxplots of each variable categorized by eight WX levels: None, HZ (haze), DU (dust), BR (mist), FG (fog), DZ (drizzle), RA (rain), and SN (snow) in order. Units are as follows; CPM (μg m^−3^), PM_2.5_ (μg m^−3^), TMP (℃), RH (%), WS (kt^*^), and VIS (km) respectively. *1 kt = 0.5144 m s^−1^.

The relationships between all variables are shown in Fig. 2, with the RH exhibiting the highest correlation with visibility (VIS), − 0.59, followed by the PM_2.5_, − 0.50, while the wind speed (WS) shows weak positive correlations of 0.15. Although the CPM displays very weak correlations of − 0.08, its correlation of − 0.21 and − 0.33 in haze and dust, respectively, at the ICN cannot be ignored (Fig. S-2). The temperature (TMP) and VIS display essentially no correlation, with values of − 0.01 at the ICN. As the most important variable, high RH significantly reduces visibility and contributes to hydrometeors formation such as fog^2^. Even in fine aerosols, the extinction coefficient values of high hygroscopicity particles increase through moisture absorption, further supporting RH as a major factor responsible for decreased visibility^21,22^. This validates the conclusion that PM_2.5_ commonly displays strong correlation with visibility^15,17,24^. According to the Mie-scattering theory, the scattering efficiency associated with the light wavelength is frequently high for particles less than 2.5 µm in diameter^40–42^. Among particles with high extinction efficiency values, sulfates and nitrates characterized by high hygroscopicity also fall in the less than 2.5 µm category^24,43^. According to previous studies, the PM_10_ contributes to visibility deterioration^11–13^, which can be attributed mostly to the PM_2.5_. Therefore, better prediction of the distribution and concentration of the fine PM is necessary to clearly understand how PMs and weather factors affect visibility.

Regarding the PM, the CPM and PM_2.5_ show very weak negative correlations with the TMP, while the RH and WS display opposite correlations, respectively. For example, the correlation between the RH and CPM is negative, while that between the RH and PM_2.5_ is positive. In the RH–CPM and RH–PM_2.5_ plots, the RH shows higher correlation with the PM_2.5_ than with the CPM (0.20 and –0.10, respectively). In the WS–PM_2.5_ plot, the lower the wind speed, the higher the concentration (− 0.20). Conversely, in the WS–CPM plot, high concentrations are observed in the case of winds exceeding 10 kt. This is explained by the negative correlations in the RH–WS plot (− 0.27), demonstrating that low wind speed and high humidity values are related to high PM_2.5_ concentrations. Since these conditions are common under stable atmospheric conditions, several studies have revealed their association with PM_2.5_ stagnation^18,45,46^. In the WX–CPM box plot, the CPM commonly displays high concentrations in dust, while in the WX–PM_2.5_ box plot, the highest PM_2.5_ concentrations occur in haze and dust. The dust-pollution is mainly influenced by the Asian Dust in spring, and the high PM_2.5_ concentration in dust indicates that in addition to the CPM, fine PM are transported into the region^47^.

### Impacts of PM and meteorological factors on visibility

The visibility prediction model determined the effects on visibility considering the PM_2.5_ and CPM variables for each weather factor (Table S-7). The contribution of each PM concentration on visibility degradation depends on the coefficient of TMP, RH, and WS. For example, the coefficients of the interactions between the PM_2.5_ and meteorological variables, TMP, RH, and WS (− 0.231, 0.676, and 0.086, respectively) show that visibility decrease at any PM_2.5_ concentration is associated with high TMP, low RH, and low WS. Conversely, the effect of any CPM concentration (0.020, 0.193, and − 0.023, respectively) is related to low TMP, low RH, and high WS. The coefficient of the PM_2.5_–TMP interactions (− 0.231) and that of the PM_2.5_–RH interactions (+ 0.676) demonstrate involvement in visibility degradation. When comparing the visibility estimation coefficient for each variable, the effect of the $Z_{RH}$, with its coefficient of –2.88 km is the highest (refer to Table S-6g). Thus, when the RH increases, the effect of the PM is reduced, while that of the RH is maintained. Conversely, at low RH, the PM mainly control the visibility impairment. For example, assuming standardized values of 0 for the TMP, RH, and WS, for haze at the ICN, the coefficient for the PM_2.5_ ($\beta_{{Z_{{PM_{2.5} }} }}$) changes to − 0.784 and that for the CPM ($\beta_{{Z_{CPM} }}$) to − 0.637. Evidently, the values for the PM_2.5_ and CPM are not significantly different, but since days with high PM concentrations are often dry ($Z_{RH} < 0$), at $Z_{RH} = - 1.0$ (RH 44%) with $Z_{TMP} = 0$ and $Z_{WS} = 0$, the effect of PM_2.5_ is − 1.46 km, while the CPM effect is − 0.83 km. Contrarily, for fog, since the humidity is very high ($Z_{RH} \gg 0$), the effect of PM_2.5_ is very low. At $Z_{RH} = + 1.6$ (RH 91%), the effect of PM_2.5_ at the ICN is − 0.02 km (− 1.098 + 0.676 * 1.6). Therefore, these relationships suggest that the visibility in fog is mainly determined by RH and influenced by the PM–RH interaction.

### Hygroscopic effects of PM

**Figure 3:**
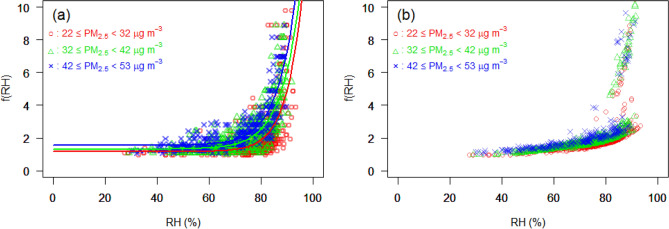
Visibility-based hygroscopic growth factor, i.e., $f\left( {RH} \right)$, of PM_2.5_ for the ICN data set in 2019, calculated by (**a**) observed and (**b**) modeled visibility, as a function of relative humidity (RH). Red circles: PM_2.5_ concentrations in the range of 22–32 μg m^−3^; green triangles: PM_2.5_ range of 32–42 μg m^−3^; blue crosses: PM_2.5_ range of 42–53 μg m^−3^. The mean and 97.5th of PM_2.5_ concentrations are indicated by 22 and 53 μg m^−3^, respectively. The modeled visibility was induced by Model 2 (Table S-2).

As mentioned in the previous section, the PM_2.5_ effect on visibility is highly dependent on the RH. Hygroscopic growth factors of PM_2.5_ for the ICN data set in 2019 are plotted in Fig. 3. The ratio between dry and wet scattering coefficients as a function of RH was calculated by observed (Fig. 3a) and modeled (Fig. 3b) visibility for three levels of PM_2.5_ concentrations. The plots show that the higher the PM_2.5_ concentration, the greater the increase in $f\left( {RH} \right)$. The $f\left( {RH} \right)$ curve shows a minor increase in $f\left( {RH} \right)$ in the RH range of 50–70% and a more continuous increase in the 70–80% range. RH > 80% leads to a rapid increase in $f\left( {RH} \right)$. These results are somewhat similar to the hygroscopic growth factor curves of PM samples obtained by other groups^10,21,22^. For the modeled $f\left( {RH} \right)$, the $f\left( {RH} \right)$ was less varied than for the observed $f\left( {RH} \right)$. Furthermore, a discontinuity in growth was observed in the 80–85% RH range. This discrepancy may relate to the visibility during fog being distinguishably lower than in other weather conditions. However, in general, the modeled $f\left( {RH} \right)$ steadily increased as RH increased (when RH < 80%). This is in good agreement with the observed $f\left( {RH} \right)$.

**Figure 4:**
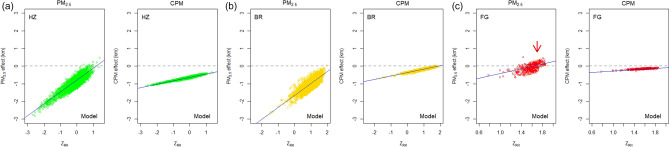
PM_2.5_ and CPM effects on visibility under haze (HZ), mist (BR), and fog (FG) from Model 6 for the ICN during the four years (2015–2018). The plots show the effect of a specific PM concentration decreases towards zero as the RH increases, with the effect of the PM_2.5_ on visibility significantly greater than that for the CPM. For fog, the effect of PM_2.5_ on visibility exhibits positive values (red arrow) at *Z*_*RH*_ values > 1.5 (RH = 89%).

Figure 4 is a plot of the effects of specific PM concentrations on visibility under haze, mist, and fog. The standardized RH is represented on the horizontal axis, and the vertical axis represents the visibility prediction at specific concentrations of the standardized CPM and PM_2.5_. According to the data in Fig. 4, as the RH increases, the effect of a specific PM concentration decreases towards zero, with an obvious linearity and change rate. Considering weather conditions, the effect of the PM_2.5_ on visibility is significantly greater than that of the CPM for haze and mist, but very low for fog. For haze, at $Z_{RH}$ of − 2 (RH 26%), the effect of specific PM_2.5_ ($Z_{{PM_{2.5} }} = 1$; PM_2.5_ = 15 μg m^−3^) increase on visibility is about − 2.3 km. Conversely, the effect a specific CPM ($Z_{CPM} = 1$; CPM = 28 μg m^−3^) increase on visibility is about − 1.0 km. As the humidity increases, the effect of specific PM on visibility decreases, nearly reaching zero when $Z_{RH} > 1$ (RH > 80%), implying dependence on RH. These characteristics depending on humidity clearly demonstrate the hygroscopicity of the particles. Apparently, without the influence of the PM and humidity interaction on the hygroscopicity, the effect on visibility of the PM_2.5_ would stay constant. However, as the humidity rises, the hygroscopic PM_2.5_ further increases the scattering efficiency, causing low visibility. The higher the RH, the greater the increase in the hygroscopic growth factor^21,22^; therefore, the visibility effect of the PM_2.5_ at high RH gradually approaches zero. Therefore, as shown in Fig. 4, the visibility reduction under a specific PM mass concentration decreases with rising humidity. This also demonstrates the high accuracy of the instrument used for the PM measurements at the Unseo station. To maintain the measurement performance considering the hygroscopic effects of the PM, a certified PM monitoring instrument requires an air condition of RH 30–40% and 20–23 ℃ for the sampled air after passing the inlet^37,48,49^. From Fig. 4, the PM effect near zero on visibility at high humidity shows that the sampled PM was properly adjusted to a dry state in the PM monitoring instrument. Such a hygroscopic effect can be utilized to improve the accuracy of any low-cost PM sensor using the light scattering principle. The field testing using the low-cost sensors considering the hygroscopic growth is presented in the next section.

Another notable observation from Fig. 4 is the characteristic of the effect of the PM on visibility in fog. In theory, the PM_2.5_ always negatively affects visibility through light scattering. However, the effect of PM_2.5_ on visibility exhibits positive values at *Z*_*RH*_ > 1.5 (RH 89%) for fog. However, these do not indicate that the PM positively impacts visibility because statistical models only describe data, instead of causal relationships. Therefore, this positive relationship is inferred to be possible if the PM_2.5_ concentration is lowered when visibility is lowered by fog. Further, the composition of the PM_2.5_ varies depending on the region. In fact, secondary products largely account for the PM_2.5_ proportion, and the SMA provides a significant amount of the secondary products such as the hydrophilic sulfates and nitrates^50–52^. According to previous studies, the hydrophilic PM_2.5_ in fog conditions grows in particle size with rising moisture or is removed by scavenging, resulting in its lower concentration^22,53^. The PM_2.5_-containing fog droplets can be inferred to increase the scattering coefficient by increasing the refraction index, and thus contribute to visibility degradation^54,55^.

**Figure 5:**
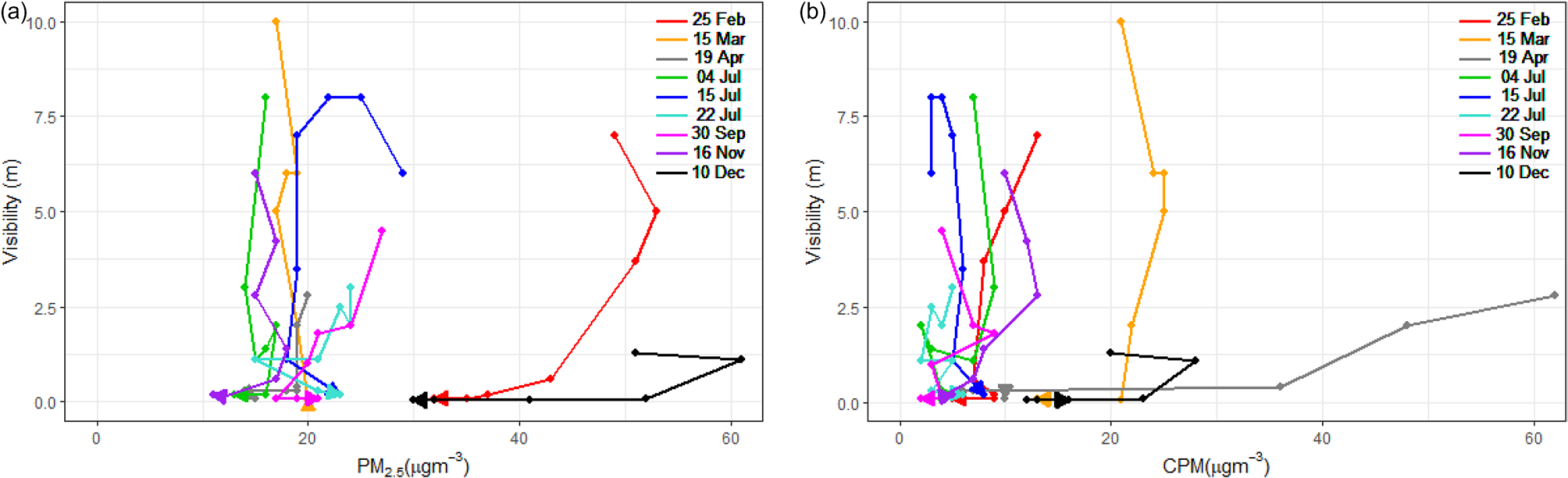
Changes in visibility and PM_2.5_ and CPM concentrations over 6 h during dense fog events for 9 of the foggiest days at the ICN in 2019. A decrease in PM_2.5_ and CPM concentrations with decreasing visibility under high PM concentrations is demonstrated.

As previously stated, in the dry state, the PM effect on visibility is negative, that is, the increase in the PM and the decrease in visibility are plausibly strongly related. However, differentiating the relationships under high RH conditions is difficult. Figure 5 shows the changes in visibility and PM_2.5_ and CPM concentration over 6 h during fog events for 9 of the most foggy days at the ICN in 2019. The cases are characterized by decreasing PM_2.5_ and CPM concentrations with decreasing visibility under high PM concentrations. As depicted in the fog chart in Fig. 4, at RH > 89% (*Z*_*RH*_ > 1.5), the effects on the visibility of the PM_2.5_ appear to be positive. This proportional relationship differs distinctly from the case of haze, which shows the negative relationship between PM concentration and visibility. Previous studies showed that aerosol loading can activate fog generation in supersaturated conditions, characterized by smaller droplets and a higher number of particles compared to clear days^2,56–58^. Therefore, lowering the PM concentration can be inferred to reduce visibility in either case. This explains the visibility degradation in fog and haze on any day with high fine PM concentration.

**Figure 6:**
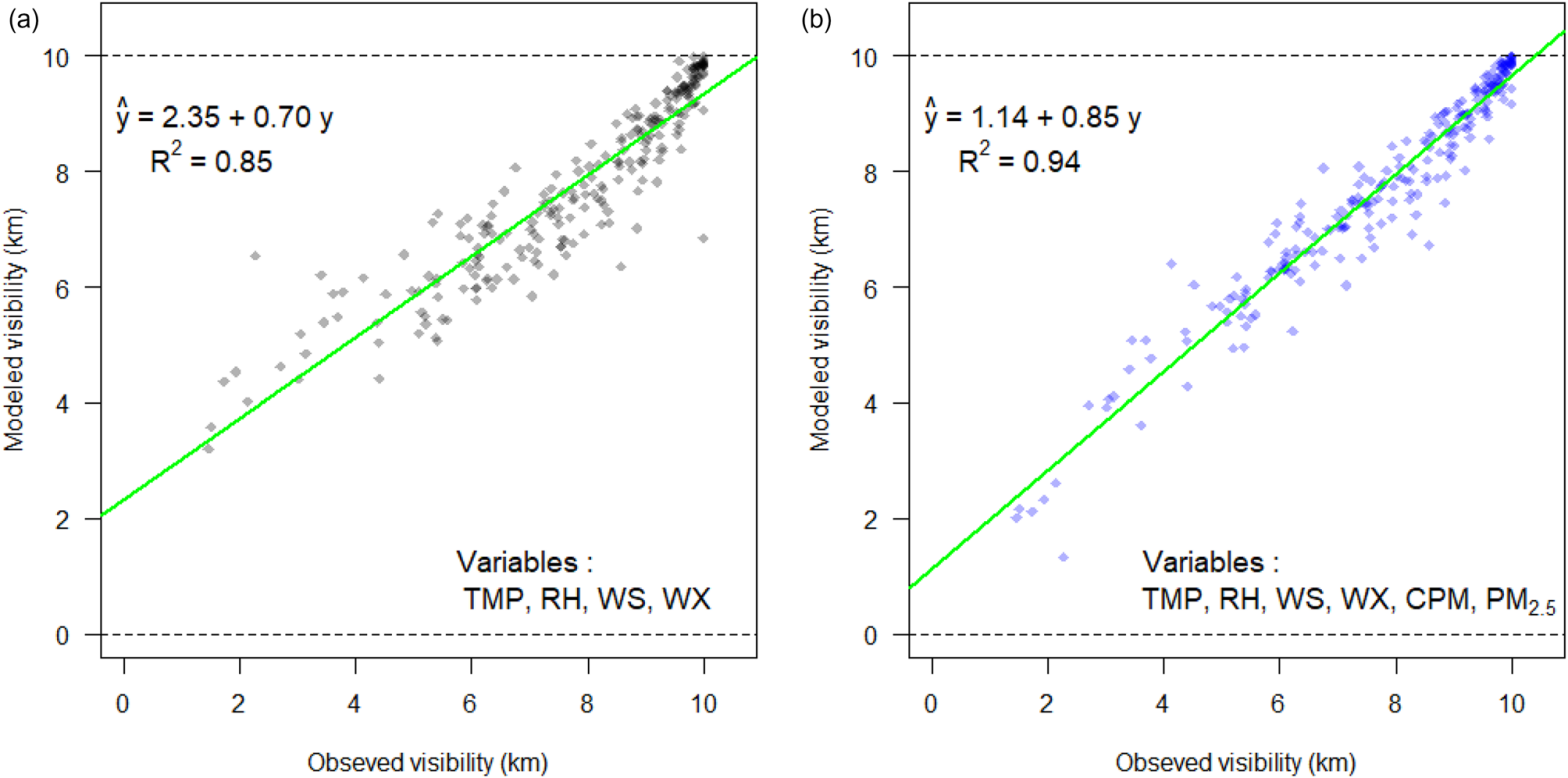
Daily averages of the modeled and observed hourly visibility from Models 0 and 6 at the ICN in 2019 (for < 10 km of modeled visibility). Model 6 has decreased interception (1.14 km) and more improved coefficient of determination (0.94) than Model 0 (Results from the other models are shown in Fig. S-3).

Figure 6 shows the daily averages of the predicted hourly visibility from a model without fine and coarse PM incorporation and another including the PM_2.5_, CPM, and their interactions. In the PM-considered model, the intercept and the coefficient of determination improved. This result shows that when the relationships between the PM concentration and meteorological factors are well understood, the visibility change associated with the PM can be better predicted. Conversely, the relationship between light scattering and the PM concentration can also be estimated by predicting visibility.

### RH-adjusted low-cost PM_2.5_sensors

**Figure 7:**
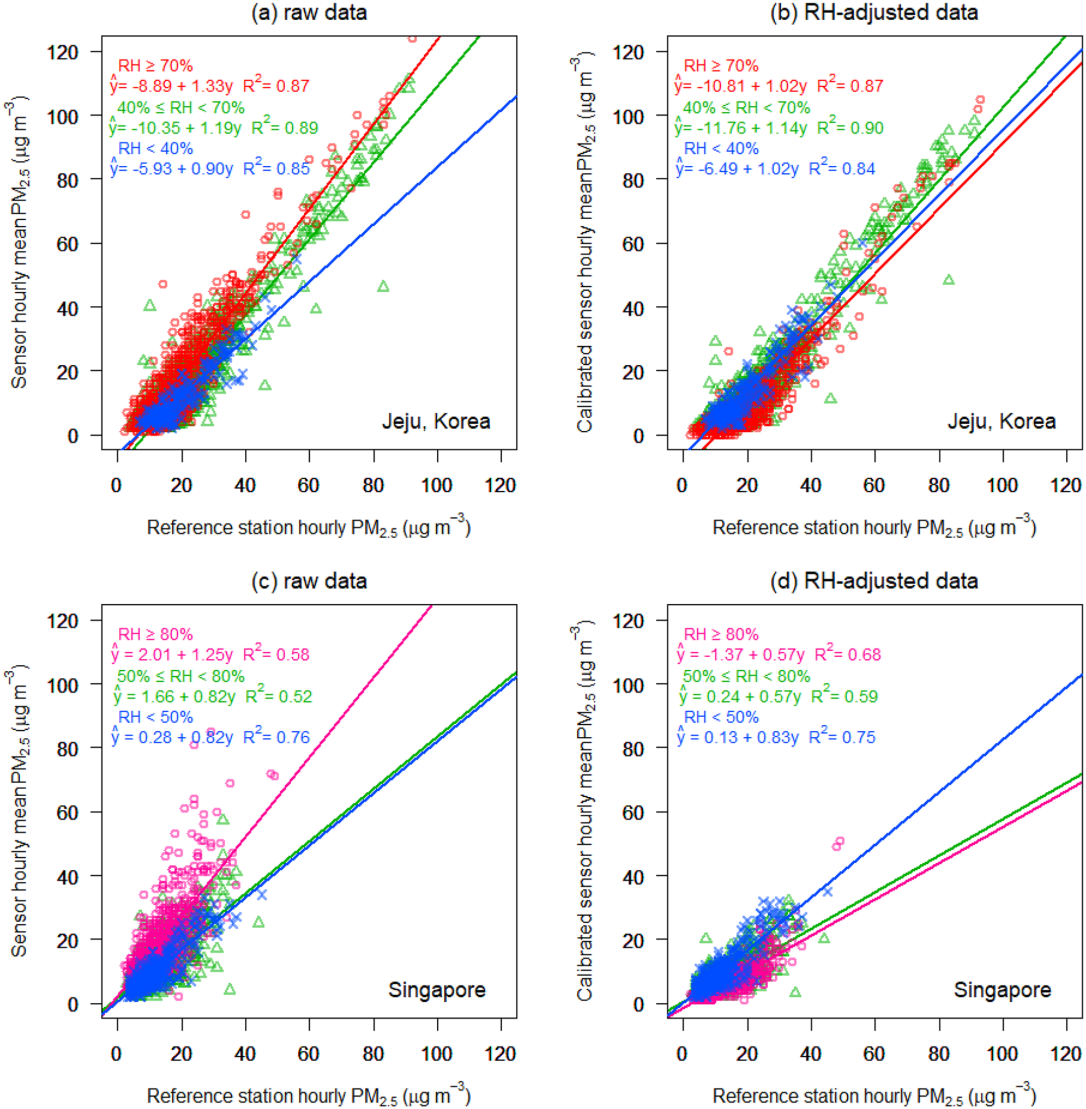
Field measurements using the low-cost PM_2.5_ sensor in Jeju, Korea and Singapore: the field testing was conducted for seven and four months in Jeju and Singapore, respectively. The plots indicate ambient PM_2.5_ concentrations measured using the low-cost PM_2.5_ sensor compared to that from the reference station. (**a**) and (**c**) show raw data, (**b**) and (**d**) show RH-adjusted data using the visibility-derived mass growth. Visibility was predicted during the field measurement by the model proposed in the present study and used to correct PM_2.5_ concentration by the visibility-derived hygroscopic mass growth proposed by Molnár et al.^59^.

A field test was conducted using the low-cost sensor in two different regions in Jeju, Korea, and Singapore for seven and four months respectively (Fig. S-5) to assess the effect of the RH on PM measurement. The raw and RH-adjusted ambient concentrations of PM_2.5_ measured using the low-cost PM_2.5_ sensor against those of the reference station are plotted in Fig. 7 for comparison. The raw data in Jeju has a negative zero drift while little or no drift was observed in Singapore. The difference in zero–drift tendency between Jeju and Singapore may be because the RH in Jeju is significantly less than that in Singapore. The raw data of the low-cost sensor in both Jeju and Singapore show a positive sensitivity under high RH (linear regression slopes of 1.33 and 1.25, respectively). On the other hand, the RH-adjusted data for the different RH ranges in Jeju exhibit reasonably good correction, and the results are in good agreement. The RH-adjusted result for high RH in Singapore shows good improvement; however, some excessive adjustment also occurs. To correct the bias of the low-cost sensor with respect to RH, the visibility-derived hygroscopic mass growth^59^ was applied to the RH-adjustment process, and the visibility was predicted by the model proposed in this study.

## Discussion

Accurately estimating the effects of PM on visibility due to the interactions between PM and weather factors is challenging. This is because low visibility is dependent on the aerosol type and properties, which are affected by hygroscopicity, wind, temperature, and season. The PM_2.5_ affects visibility significantly more than the CPM, but in weather conditions such as dust and haze as well as low RH, the CPM effects are sometimes not negligible. Increasing wind speed is associated with decreasing PM_2.5_ concentration and visibility recovery, whereas the CPM exhibits a relationship in which its concentration increases with wind speed^46^. This is also related to the generation and transport mechanisms of the PM_10_, as revealed by previous studies^45,60^. Regarding the relationship between the PM concentration and meteorological variables, the CPM exhibits negative and positive correlations with the RH and WS, respectively, whereas the corresponding PM_2.5_ relationships are positive and negative. Alternatively, this means that the CPM is associated with low RH and higher WS, while high PM_2.5_ concentration is related to high RH and low WS. As such, the weather conditions involving high PM_2.5_ concentrations resemble those of fog generation, explaining the development of haze and haze-fog from high PM_2.5_ concentrations^7^. The similarity of the weather conditions causing low visibility and high PM_2.5_ concentrations means that under certain conditions, attention to the synergy effect that may worsen visibility is necessary.

The optical hygroscopic growth of the scattering coefficient, confirmed by several studies, is prominent in PM_2.5_ with increasing RH. We calculated the hygroscopic growth factor based on the observed and modeled visibility. The results revealed that the modeled $f\left( {RH} \right)$ agreed well with the observed $f\left( {RH} \right)$ at < 80% RH, which is mostly in the RH range of the haze and mist containing PM_2.5_. Previous studies on visibility suggest that RH of 90% or higher were excluded considering the effect of particle growth^12,61,62^ or the effect of the PM on visibility is insensitive at RH > 90%^17,18^. However, we demonstrated variation of the effect of the PM on visibility over the entire RH range. Regarding visibility reduction, the fact that the visibility change at high RH is insensitive to the PM_2.5_ concentration does not imply negligible influence by the PM_2.5_. Considering PM_2.5_ and RH as independent variables, the effect of RH is relatively higher as the RH increases.

As shown in Fig. 4, in most cases, regardless of the weather conditions, an increase in the PM mass concentration is associated with a decrease in visibility, with the variation controlled by parameters such as TMP, RH, and WS. However, for fog associated with high RH, the visibility impact of RH is very high, while that of the PM_2.5_ is relatively very weak, thereby rendering differentiation of the PM effect difficult. Rather, the positive correlation of the influence of PM_2.5_ and visibility can be considered a particle scavenging phenomenon through which the PM_2.5_ decreases as the visibility decreases^53^. The decrease in the PM mass concentration with increasing RH or persisting fog due to interaction with moisture signifies loss of extinction ability by the PM_2.5_. Owing to the inverse relationship between PM_2.5_ and RH under foggy conditions, quantifying the effect of PM on visibility at high RH is difficult. However, the PM_2.5_ concentration decrease is explained by interaction with water droplets. Previous studies suggested that the higher the PM_2.5_, the higher concentration of condensation nuclei, and thus the droplets increase; consequently, fog formation is activated and the dissolution of PM in the water droplet increases the refractive indexes^2,55,63,64^. Therefore, an increase in PM can cause visibility degradation in different situations. Alternatively, the PM_2.5_ concentration decrease is adequately related to the visibility decrease. This relation demonstrates that the visibility is generally inversely proportional to the PM_2.5_ concentration, whereas the relationship is proportional in fog.

Further, to assess the low-cost PM sensors with RH-adjusted correction, we conducted field measurements at two different locations (Fig. S-5). We found that the RH-adjusted PM_2.5_ concentration data based on the visibility-derived hygroscopic mass growth exhibit a reduced bias, which is dependent on RH. The results suggest that the observation that PM affects visibility by interacting with RH and other meteorological factors can be applied to PM sensors using the principle of light scattering. Recently, low-cost PM sensors are increasingly being used, but their accuracies are significantly lower than those of certified instruments unless meteorological conditions are effectively controlled^65,66^. Instead, field calibration methods have been proposed for improving sensor accuracy by considering the effects of regional and environmental conditions^65,67,68^.

Although many studies have suggested PM sensor accuracy improvement by self-calibration, controversy still remains^65,66^. According to some studies, visibility and temperature are not significantly related, but long-term measurements may yield different results, and different calibrations exhibit significance depending on the regional environmental and climate conditions^28,32,33,67,69^. Johnson et al.^28^ showed accuracy variations for the same sensor according to climatic conditions of cities and countries, while Zusman et al.^67^ demonstrated the effectiveness of different calibration methods for different regions. We collected data spanning four years from the airport, developed the visibility model, and applied the results to PM_2.5_ monitoring using the low-cost sensor. The results may not provide enough evidence of a causal effect between CPM and PM_2.5_ and weather factors. However, the present study provides valuable data for predicting visibility at airports and in the transportation industry and, most importantly, suggests that the findings can be used to improve the performance of low-cost PM sensors. As visibility data are abundant and available worldwide, future work should include various region-specific experiments in different environments for easy and beneficial ambient PM monitoring using low-cost sensors.

## Methods

### Data

To investigate the effect of PM on visibility and the associated meteorological variables, meteorological data were collected at the ICN over a four period (2015–2018). This involved collecting PM_2.5_ and PM_10_ concentrations data from the Unseo air-quality monitoring stations. The PM concentrations at the Unseo station located 5 km northeast of the ICN (Fig. S-1), were assumed to be representative of the ICN. Over the four years, continuous data for 35,064 h were collected at the ICN. The data included the wind direction, wind speed, visibility, *present weather*^35^, temperature, and dew-point temperature, with the RH calculated from the temperature and dew-point temperature^70^. The ‘*present weather*’ was separated into eight major categories such as the HZ, DU, BR, FG, DZ, RA, SN, and None, according to the WMO guidelines^35,39^. (The two-letter abbreviations and the explanation of the *present weather* are provided in Tables S-3, 4) The CPM concentration was calculated as the difference between the PM_2.5_ and PM_10_ concentration. If either PM_10_ or PM_2.5_ was missing, this was treated as missing, yielding 29,321 (missing 5743, missing rate 19.59%) hours of data for the Unseo Station.

The weather observations and PM data were standardized to have mean zero and variance 1 to assess the influence of each variable. In the present study, the standardized variable is utilized because the goal is to evaluate the interaction of each variable with PM and the corresponding influence on visibility, rather than predicting visibility from a single variable. The variables and the corresponding standardized data are presented in Table S-1. The standardized variable $Z_{{x_{i} }}$ is determined as follows:

1 $$Z_{{x_{i} }} = \frac{{x_{i} - \overline{{x_{i} }} }}{{s_{{x_{i} }} }}$$

where $x_{i}$ is the meteorological variable and PM concentration, $Z_{{x_{i} }}$ is the standardized variable of $x_{i}$, $\overline{{x_{i} }}$ is the mean value of the variables, and $s_{{x_{i} }}$ is the standard deviation of $x_{i}$.

### Model design

The Tobit model, representing a censored regression, was employed for evaluating the effects of the meteorological variables and PM concentration on visibility^46^. Unlike the conventional regression model, the censored regression model involves data with a limited range with incomplete information. The data (i.e., explanatory variable) have an upper or lower limit which is beyond the boundaries; however, their extent above or below the boundary remains unknown (e.g., an observed time from an individual still alive in the survival analysis). Since the visibility observation at the airport involves an upper limit (9999 m)^25^, the data display censored characteristics expressed as follows:

2 $$y_{i}^{*} = Z_{{x_{i} }} \beta + \varepsilon_{i}$$

3 $$y_{i} = \left\{ {\begin{array}{*{20}l} l \hfill & {(y_{i}^{*} \le l)} \hfill \\ {y_{i}^{*} } \hfill & {(l < y_{i}^{*} < u)} \hfill \\ u \hfill & {(y_{i}^{*} \ge u)} \hfill \\ \end{array} } \right.$$

where $y_{i}$ is the airport visibility, $\beta$ is the slope of the visibility for the standardized variable reflecting the visibility prediction coefficient, $l$ is the lower censoring value (0 m), u is the upper censoring value (9999 m), and $\varepsilon_{i} { }\sim { }N\left( {0,\sigma^{2} } \right)$ is a random error.

Owing to the weak correlations between the variables shown in Fig. 2 and the low variance inflation factor values in Table S-5, multicollinearity can be ignored in this study. Seven models (Model 0–6) were constructed from the data in Table S-2, to assess the effects of the PM_2.5_, CPM, and interaction term on visibility. The PM_2.5_ and CPM were excluded for Model 0, while the PM_2.5_ was included in Models 1–6, with Models 2–6 designed with or without the CPM and its interaction term with the weather variables. The odd-numbered models represent a “reduced model” of even-numbered models lacking interaction terms between the PM_2.5_ and meteorological variables. The visibility estimation coefficients and statistics for each model are provided in Table S-6. The fitting results for different weather variables and models are presented in Fig. S-3. Ideally, the intercept of the model should coincide with the origin (0), while the coefficient of determination $R^{2}$ close to unity (1). Under these conditions, the linearity for Models 2, 4, and 6 were better than those for Models 1, 3, and 5. This means that Models 2, 4, and 6 involving the PM_2.5_ interactions are more adequate for predicting low visibility. Although all models were suitable for predicting visibility, Model 6, the most appropriate and valid for comprehensively analyzing the effects of PM_2.5_ and CPM was adopted in the present study because it also has the minimum Akaike information criterion (Table S-2). According to the models from ICN, the mean squared error of Model 6 is smallest among 7 models for most weather conditions with high RH values, such as BR, FG, DZ, RA, and SN (Table S-8). The most suitable model, however, can be selected based on the availability of variables, with Model 2 observed to be most effective when the PM_10_ was unavailable.

### Field measurement of PM_2.5_and RH-adjustment

The field measurement of PM_2.5_ was conducted using a low-cost sensor, namely the *W-station* (Observer Co.), which includes a particulate-matter sensor, *SPS30* (Sensirion), which uses laser-based light scattering (specifications of the sensors and locations of the field measurement are provided in Table S-9 and Fig. S-5). The sensor transmits PM_2.5_ mass concentration with temperature and humidity every 300 s via Wi-Fi and does not have air-conditioning functions. The RH-adjusted value was calculated using the visibility-derived hygroscopic mass growth developed by Molnár, et al.^59^ as follows:

4 $$f\left( {RH} \right) = \frac{{\sigma_{ext} \left( {RH} \right)}}{{\sigma_{ext} \left( {dry} \right)}} = \frac{{VIS\left( {dry} \right)}}{{VIS\left( {RH} \right)}}$$

5 $$GM_{VIS} = f\left( {RH} \right)^{\frac{3}{2}}$$

6 $$GM_{filter} = GM_{VIS} \times \left( {1.1703 - 0.0076 \times RH} \right)$$

7 $$m_{{PM_{2.5} }} \left( {dry} \right) = \frac{{m_{{PM_{2.5} }} \left( {RH} \right)}}{{GM_{filter} }}$$

where $f\left( {RH} \right)$ is the aerosol optical hygroscopic growth factor, the ratio of the extinction coefficient at a given RH to that in dry condition; $\sigma_{ext}$ is the total extinction coefficient at a wavelength of 550 nm calculated by visibility (VIS); $GM_{VIS}$ is the mass growth rate calculated by the optical growth factor; $GM_{filter}$ is the mass growth rate calculated by the gravimetric method, weighing the mass change of aerosol filters owing to RH variation; and $m_{{PM_{2.5} }} \left( {dry} \right)$ is the RH-adjusted mass concentration obtained using the low-cost PM sensor. The relationship between $GM_{VIS}$ and $f\left( {RH} \right)$ is derived considering they correspond to the cross-sectional area and the volume of the particles, respectively. The dry condition corresponded to a RH of 35%, considering that RH-conditioning in the reference instrument was 30–40%.

## Supplementary Information

Supplementary Information.
