# Supplementary material for: Hygroscopic properties of particulate matter and effects of their interactions with weather on visibility
Source: Sci Rep. 2021 Aug 12;11:16401. doi: 10.1038/s41598-021-95834-6 (PMC8361198; doi:10.1038/s41598-021-95834-6)
Supplement: Supplementary file 1 — Supplementary Information. [file 41598_2021_95834_MOESM1_ESM.docx]

**Supplementary Material**

**Hygroscopic properties of particulate matter and effects of their interactions with weather on visibility**

**Wan-Sik Won^1^, Rosy Oh^2^, Woojoo Lee^3^, Sungkwan Ku^4^, Pei-Chen Su^1*^, Yong-Jin Yoon^1,5*^**

*^1^ School of Mechanical and Aerospace Engineering, Nanyang Technological University, Singapore 639798, Singapore*

*^2^ Department of Industrial and Systems Engineering, Korea Advanced Institute of Science and Technology (KAIST), Daejeon 34141, Korea*

*^3^ Department of Public Health Sciences, Graduate School of Public Health, Seoul National University, Seoul 08826, Korea*

*^4^ Department of Aviation Industrial and System Engineering, Hanseo University, Chungcheongnam-do 32158, Korea*

*^5^ Department of Mechanical Engineering, Korea Advanced Institute of Science and Technology (KAIST), Daejeon 34141, Korea*


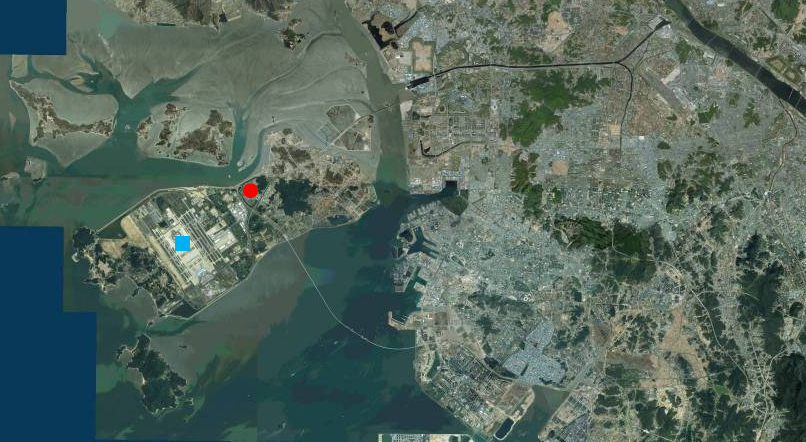


Map data ©2020 NGII

Fig. S-1. Location of Incheon International Airport (ICN) (blue square) and Unseo air quality monitoring station (red circle). Map data from National Geographic Information Institute, Korea (NGII, 2020).

^*^ICN: IATA 3-letter code for Incheon International Airport; IATA stands for the International Air Transport Association.

Table S-1. Data summary of the variables^a^ collected at the ICN and Unseo station, 2015–2018: $Z_{CPM}$, $Z_{{PM}_{2.5}}$, $Z_{TMP}$, $Z_{RH}$, and $Z_{WS}$, are standardized parameters of $CPM$, ${PM}_{2.5}$, $TMP$, $RH$, and $WS$, respectively.

|  | $VIS$ (km) | $CPM$  (μg m^-3^) | $Z_{CPM}$ | ${PM}_{2.5}$  (μg m^-3^) | $Z_{{PM}_{2.5}}$ | $TMP$ (℃) | $Z_{TMP}$ | $RH$ (%) | $Z_{RH}$ | $WS$ (kt)^b^ | $Z_{WS}$ |
| --- | --- | --- | --- | --- | --- | --- | --- | --- | --- | --- | --- |
| Min. | 0.05 | 0 | -0.75 | 1 | -1.48 | -16 | -2.68 | 10 | -2.88 | 0 | -1.72 |
| 1st Qu. | 7.00 | 10 | -0.40 | 12 | -0.73 | 3 | -0.82 | 48 | -0.75 | 4 | -0.79 |
| Median | 10.00 | 16 | -0.19 | 20 | -0.19 | 12 | 0.03 | 63 | 0.06 | 7 | -0.09 |
| Mean | 8.37 | 21 | 0.00 | 23 | 0.00 | 12 | 0.00 | 62 | 0.00 | 7 | 0.00 |
| 3rd Qu. | 10.00 | 26 | 0.16 | 30 | 0.48 | 21 | 0.84 | 77 | 0.84 | 10 | 0.61 |
| Max. | 10.00 | 911 | 31.39 | 159 | 9.20 | 37 | 2.38 | 98 | 1.98 | 32 | 5.73 |

^a^ abbreviation; VIS (Visibility), CPM (Coarse particulate matter), TMP (Temperature), RH (Relative humidity), WS (Wind speed)
^b^ 1 kt = 0.5144 m s^-1^

Table S-2. Variables applied to model design, degrees of freedom (df), and Akaike information criterion (AIC) from each model. Plus (+) signs indicate when the variable is incorporated in the model and minus (-) signs indicate when it is not.

| **Model**  **No.** | TMP | RH | WS | WX^a^ | PM_2.5_ | CPM | PM_2.5_ interactions | CPM interactions | df | AIC^b^  (ICN) |
| --- | --- | --- | --- | --- | --- | --- | --- | --- | --- | --- |
| 0 | + | + | + | + | – | – | – | – | 12 | 43761 |
| 1 | + | + | + | + | + | – | – | – | 13 | 41243 |
| 2 | + | + | + | + | + | – | + | – | 23 | **40367** |
| 3 | + | + | + | + | + | + | – | – | 14 | 41142 |
| 4 | + | + | + | + | + | + | + | – | 24 | **40254** |
| 5 | + | + | + | + | + | + | – | + | 24 | 40885 |
| 6 | + | + | + | + | + | + | + | + | 34 | **40097** |

^*^WX (weather) has 8 levels: None, FG (fog), BR (mist), HZ (haze), DU (dust), DZ (drizzle), RA (rain), and SN (snow), which are categorical variables of 0 or 1.

| **Haze (HZ)**  **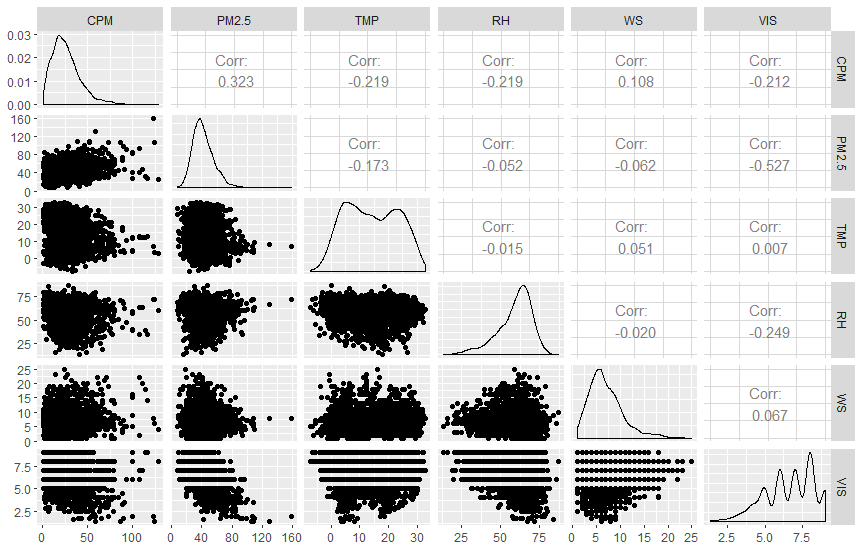** | **Dust (DU)**  **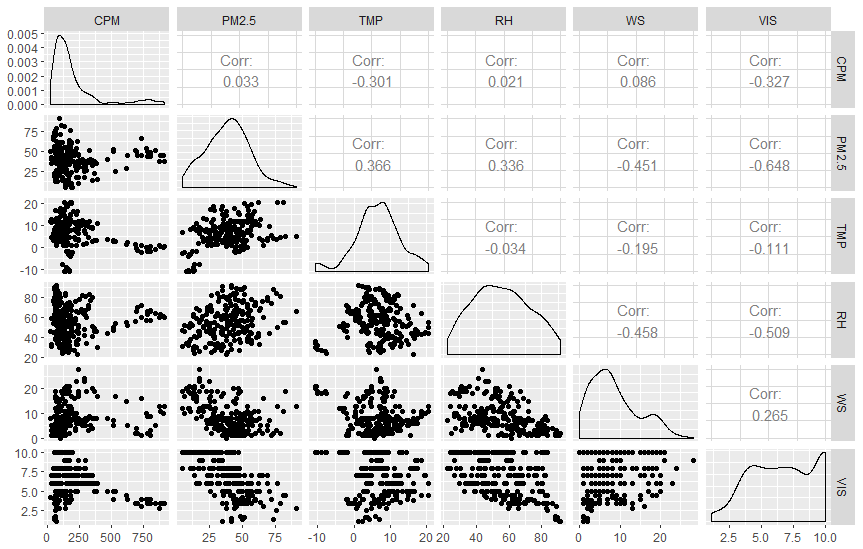** |
| --- | --- |
| **Mist (BR)**  **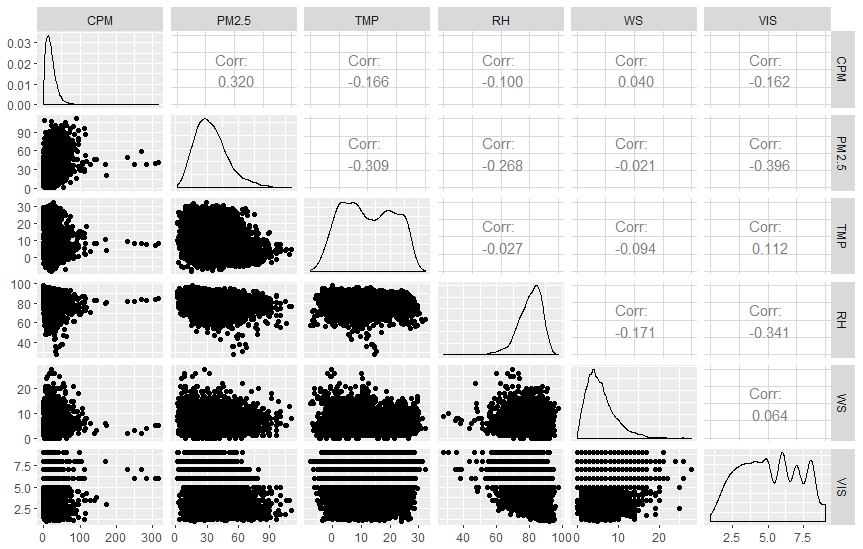** | **Fog (FG)**  **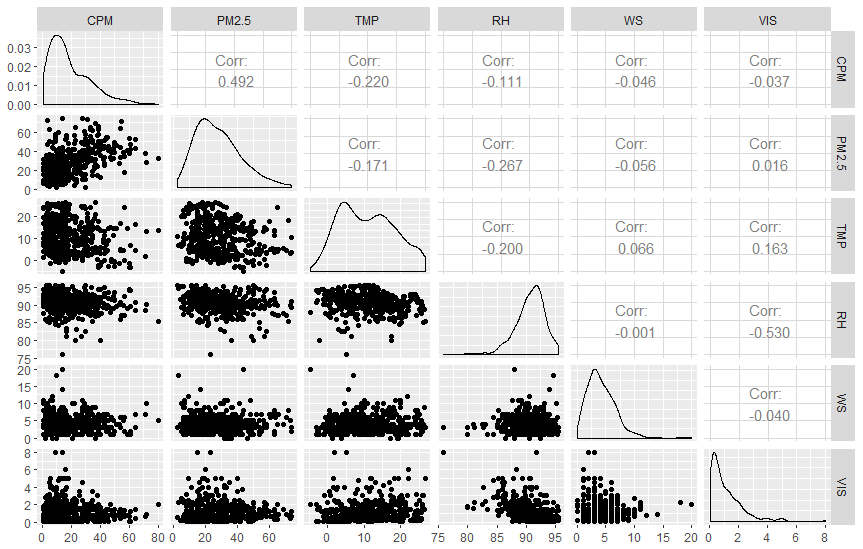** |
| **Drizzle (DZ)**  **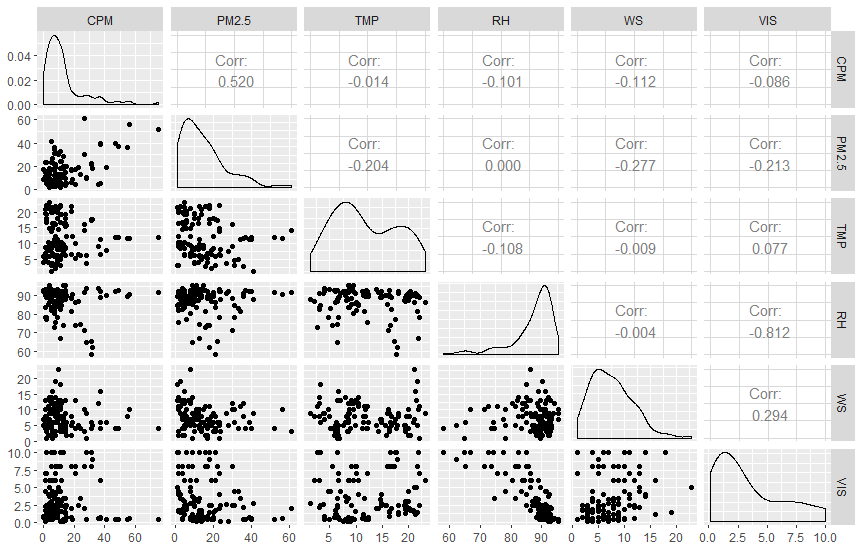** | **Rain (RA)**  **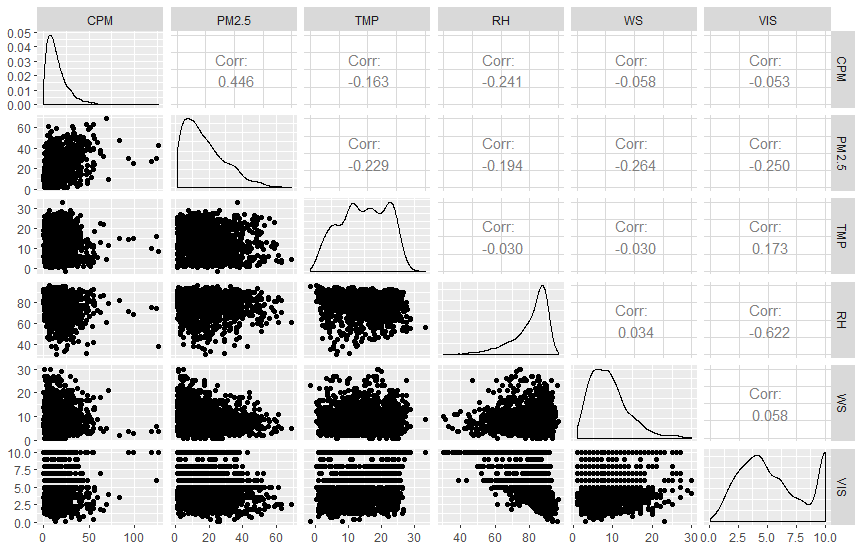** |
| **Snow (SN)**  **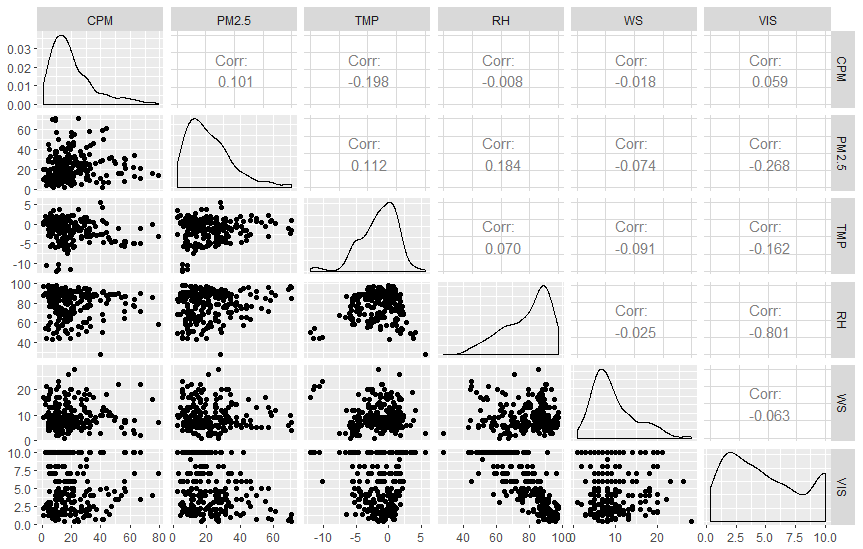** | **No significant weather (None)**  **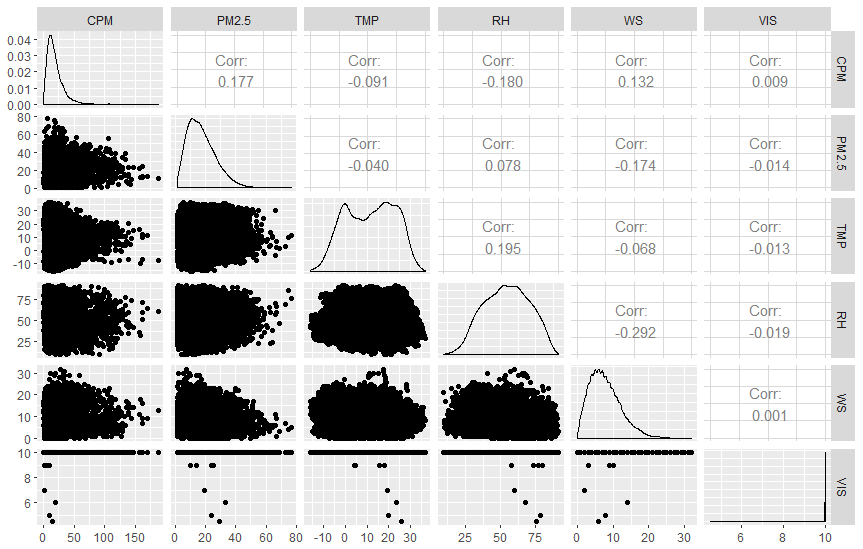** |

Fig. S-**2**. Matrix of plots and correlation coefficients between variables for each weather variable at the ICN.

Table S-3. Two-letter abbreviations of weather phenomena for significant *present weather* reported in METAR (aerodrome routine meteorological report) (WMO-No.306, 2017)

| Precipitation | | Obscuration | | Others | |
| --- | --- | --- | --- | --- | --- |
| DZ* | Drizzle | BR* | Mist | PO | Dust/sand whirls (dust devils) |
| RA* | Rain | FG* | Fog | SQ | Squalls |
| SN* | Snow | FU | Smoke | FC | Funnel cloud(s) (tornado or waterspout) |
| SG | Snow grains | VA | Volcanic ash | SS | Sandstorm |
| PL | Ice pellets | DU* | Widespread dust | DS | Duststorm |
| GR | Hail | SA | Sand |  |  |
| GS | Small hail and/or snow pellets | HZ* | Haze |  |  |
| UP | Unknown precipitation |  |  |  |  |

* 7 *present weather* is that used in this study

Table S-4. Explanation of weather phenomena significant to aviation (WMO-No.782, 2014)

| Haze (HZ) | The suspension in the air of extremely small dry particles invisible to the naked eye and sufficiently numerous to give the air an opalescent appearance with a reduction in horizontal visibility to 5,000 m or less. |
| --- | --- |
| Dust (DU) | The reduction of horizontal visibility to 5,000 m or less caused by the suspension in the air of small particles of dust raised from the ground. |
| Mist (BR) | The suspension of microscopic water droplets or wet hygroscopic particles in the air, reducing horizontal visibility to 1,000 to 5,000 m. |
| Fog (FG) | The suspension in the air of very small water droplets, which reduces horizontal visibility to less than 1,000 m. |
| Drizzle (DZ) | Fairly uniform precipitation in very fine drops of water with a diameter of less than 0.5 mm. |
| Rain (RA) | Precipitation of liquid water droplets of appreciable size (greater than 0.5 mm). |
| Snow (SN) | Solid precipitation of single or agglomerated ice crystals falling from a cloud. |

Table S-5. Multicollinearity diagnostics result of variance inflation factor at the ICN

|  | TMP | RH | WS | PM_2.5_ | CPM |
| --- | --- | --- | --- | --- | --- |
| ICN | 1.03 | 1.14 | 1.11 | 1.14 | 1.08 |

Table S-6. Estimation results of Models 0-6

1. Model 0

|  | Estimate | Std. Error | z-value | p-value^a^ |  |
| --- | --- | --- | --- | --- | --- |
| (Intercept):1 | 17.33 | 0.1959 | 88.48 | 0.0000 | *** |
| (Intercept):2 | 0.7133 | 0.007079 | 100.8 | 0.0000 | *** |
| $Z_{TMP}$ | 0.2126 | 0.02455 | 8.661 | 0.0000 | *** |
| $Z_{RH}$ | -1.844 | 0.04016 | -45.92 | 0.0000 | *** |
| $Z_{WS}$ | 0.07762 | 0.02236 | 3.471 | 0.0005 | *** |
| $HZ$ | -11.03 | 0.2011 | -54.83 | 0.0000 | *** |
| $DU$ | -10.81 | 0.2422 | -44.61 | 0.0000 | *** |
| $BR$ | -10.29 | 0.1952 | -52.75 | 0.0000 | *** |
| $FG$ | -13.22 | 0.2176 | -60.73 | 0.0000 | *** |
| $DZ$ | -11.33 | 0.2711 | -41.79 | 0.0000 | *** |
| $RA$ | -9.59 | 0.2006 | -47.8 | 0.0000 | *** |
| $SN$ | -10.15 | 0.2396 | -42.37 | 0.0000 | *** |

^a^ Signif. codes: *, **, *** Significant at the 0.05, 0.01, and 0.001 probability level, respectively.

1. Model 1

|  | Estimate | Std. Error | z-value | p-value^a^ |  |
| --- | --- | --- | --- | --- | --- |
| (Intercept):1 | 16.77 | 0.1767 | 94.88 | 0.0000 | *** |
| (Intercept):2 | 0.5891 | 0.007069 | 83.34 | 0.0000 | *** |
| $Z_{TMP}$ | -0.09778 | 0.02251 | -4.343 | 0.0000 | *** |
| $Z_{RH}$ | -2.152 | 0.03669 | -58.67 | 0.0000 | *** |
| $Z_{WS}$ | -0.04437 | 0.01996 | -2.223 | 0.0262 | * |
| $HZ$ | -9.205 | 0.1817 | -50.67 | 0.0000 | *** |
| $DU$ | -9.354 | 0.2183 | -42.85 | 0.0000 | *** |
| $BR$ | -8.71 | 0.1759 | -49.51 | 0.0000 | *** |
| $FG$ | -11.94 | 0.1953 | -61.14 | 0.0000 | *** |
| $DZ$ | -10.86 | 0.242 | -44.89 | 0.0000 | *** |
| $RA$ | -8.955 | 0.1803 | -49.67 | 0.0000 | *** |
| $SN$ | -9.712 | 0.215 | -45.16 | 0.0000 | *** |
| $Z_{{PM}_{2.5}.}$ | -0.9661 | 0.01838 | -52.56 | 0.0000 | *** |

1. Model 2

|  | Estimate | Std. Error | z-value | p-value^a^ |  |
| --- | --- | --- | --- | --- | --- |
| (Intercept):1 | 17.18 | 0.1731 | 99.23 | 0.0000 | *** |
| (Intercept):2 | 0.5516 | 0.007078 | 77.93 | 0.0000 | *** |
| $Z_{TMP}$ | 0.05709 | 0.02582 | 2.211 | 0.0270 | * |
| $Z_{RH}$ | -2.798 | 0.04519 | -61.92 | 0.0000 | *** |
| $Z_{WS}$ | -0.07764 | 0.02061 | -3.767 | 0.0002 | *** |
| $HZ$ | -9.63 | 0.1831 | -52.61 | 0.0000 | *** |
| $DU$ | -9.604 | 0.2632 | -36.5 | 0.0000 | *** |
| $BR$ | -8.415 | 0.169 | -49.81 | 0.0000 | *** |
| $FG$ | -11.64 | 0.1893 | -61.51 | 0.0000 | *** |
| $DZ$ | -10.32 | 0.2568 | -40.18 | 0.0000 | *** |
| $RA$ | -8.765 | 0.1735 | -50.52 | 0.0000 | *** |
| $SN$ | -9.342 | 0.2081 | -44.9 | 0.0000 | *** |
| $Z_{{PM}_{2.5}.}$ | -1.35 | 0.1929 | -6.996 | 0.0000 | *** |
| $Z_{TMP}:Z_{{PM}_{2.5}}$ | -0.2138 | 0.01964 | -10.88 | 0.0000 | *** |
| $Z_{RH}:Z_{{PM}_{2.5}}$ | 0.7371 | 0.03056 | 24.12 | 0.0000 | *** |
| $Z_{WS}:Z_{{PM}_{2.5}}$ | 0.06976 | 0.01608 | 4.339 | 0.0000 | *** |
| $HZ:Z_{{PM}_{2.5}}$ | 0.4567 | 0.1964 | 2.326 | 0.0200 | * |
| $DU:Z_{{PM}_{2.5}}$ | 0.1586 | 0.2271 | 0.698 | 0.4849 |  |
| $BR:Z_{{PM}_{2.5}}$ | -0.3772 | 0.1918 | -1.966 | 0.0492 | * |
| $FG:Z_{{PM}_{2.5}}$ | 0.1138 | 0.2091 | 0.544 | 0.5863 |  |
| $DZ:Z_{{PM}_{2.5}}$ | -0.5575 | 0.278 | -2.005 | 0.0449 | * |
| $RA:Z_{{PM}_{2.5}}$ | -0.7432 | 0.1978 | -3.756 | 0.0002 | *** |
| $SN:Z_{{PM}_{2.5}}$ | -0.3895 | 0.229 | -1.701 | 0.0889 | . |

1. Model 3

|  | Estimate | Std. Error | z-value | p-value^a^ |  |
| --- | --- | --- | --- | --- | --- |
| (Intercept):1 | 16.74 | 0.1754 | 95.46 | 0.0000 | *** |
| (Intercept):2 | 0.5843 | 0.007068 | 82.67 | 0.0000 | *** |
| $Z_{TMP}$ | -0.1168 | 0.02249 | -5.192 | 0.0000 | *** |
| $Z_{RH}$ | -2.17 | 0.0366 | -59.3 | 0.0000 | *** |
| $Z_{WS}$ | -0.03602 | 0.01988 | -1.811 | 0.0701 | . |
| $HZ$ | -9.183 | 0.1803 | -50.92 | 0.0000 | *** |
| $DU$ | -8.318 | 0.2385 | -34.88 | 0.0000 | *** |
| $BR$ | -8.685 | 0.1745 | -49.76 | 0.0000 | *** |
| $FG$ | -11.9 | 0.1939 | -61.4 | 0.0000 | *** |
| $DZ$ | -10.85 | 0.2405 | -45.1 | 0.0000 | *** |
| $RA$ | -8.938 | 0.1789 | -49.96 | 0.0000 | *** |
| $SN$ | -9.702 | 0.2136 | -45.42 | 0.0000 | *** |
| $Z_{{PM}_{2.5}.}$ | -0.9409 | 0.01847 | -50.95 | 0.0000 | *** |
| $Z_{CPM}$ | -0.1597 | 0.01574 | -10.14 | 0.0000 | *** |

1. Model 4

|  | Estimate | Std. Error | z-value | p-value^a^ |  |
| --- | --- | --- | --- | --- | --- |
| (Intercept):1 | 17.15 | 0.1719 | 99.77 | 0.0000 | *** |
| (Intercept):2 | 0.5461 | 0.007077 | 77.17 | 0.0000 | *** |
| $Z_{TMP}$ | 0.04158 | 0.02573 | 1.616 | 0.1061 |  |
| $Z_{RH}$ | -2.813 | 0.04501 | -62.5 | 0.0000 | *** |
| $Z_{WS}$ | -0.07 | 0.02051 | -3.413 | 0.0006 | *** |
| $HZ$ | -9.606 | 0.1818 | -52.84 | 0.0000 | *** |
| $DU$ | -8.501 | 0.2814 | -30.21 | 0.0000 | *** |
| $BR$ | -8.389 | 0.1677 | -50.02 | 0.0000 | *** |
| $FG$ | -11.61 | 0.1879 | -61.8 | 0.0000 | *** |
| $DZ$ | -10.29 | 0.2554 | -40.3 | 0.0000 | *** |
| $RA$ | -8.747 | 0.1723 | -50.78 | 0.0000 | *** |
| $SN$ | -9.325 | 0.2067 | -45.13 | 0.0000 | *** |
| $Z_{{PM}_{2.5}.}$ | -1.334 | 0.1938 | -6.883 | 0.0000 | *** |
| $Z_{CPM}$ | -0.1625 | 0.01519 | -10.7 | 0.0000 | *** |
| $Z_{TMP}:Z_{{PM}_{2.5}}$ | -0.2202 | 0.01955 | -11.26 | 0.0000 | *** |
| $Z_{RH}:Z_{{PM}_{2.5}}$ | 0.7322 | 0.03041 | 24.07 | 0.0000 | *** |
| $Z_{WS}:Z_{{PM}_{2.5}}$ | 0.07342 | 0.016 | 4.59 | 0.0000 | *** |
| $HZ:Z_{{PM}_{2.5}}$ | 0.468 | 0.1972 | 2.374 | 0.0176 | * |
| $DU:Z_{{PM}_{2.5}}$ | 0.1387 | 0.228 | 0.608 | 0.5430 |  |
| $BR:Z_{{PM}_{2.5}}$ | -0.3659 | 0.1926 | -1.899 | 0.0575 | . |
| $FG:Z_{{PM}_{2.5}}$ | 0.1459 | 0.2096 | 0.696 | 0.4866 |  |
| $DZ:Z_{{PM}_{2.5}}$ | -0.5187 | 0.2779 | -1.866 | 0.0620 | . |
| $RA:Z_{{PM}_{2.5}}$ | -0.7186 | 0.1985 | -3.619 | 0.0003 | *** |
| $SN:Z_{{PM}_{2.5}}$ | -0.3978 | 0.2293 | -1.735 | 0.0828 | . |

1. Model 5

|  | Estimate | Std. Error | z-value | p-value^a^ |  |
| --- | --- | --- | --- | --- | --- |
| (Intercept):1 | 17.38 | 0.3512 | 49.49 | 0.0000 | *** |
| (Intercept):2 | 0.5713 | 0.007086 | 80.62 | 0.0000 | *** |
| $Z_{TMP}$ | -0.1347 | 0.02232 | -6.037 | 0.0000 | *** |
| $Z_{RH}$ | -2.322 | 0.03779 | -61.45 | 0.0000 | *** |
| $Z_{WS}$ | -0.01614 | 0.01986 | -0.813 | 0.4165 |  |
| $HZ$ | -9.737 | 0.3535 | -27.54 | 0.0000 | *** |
| $DU$ | -8.476 | 0.3983 | -21.28 | 0.0000 | *** |
| $BR$ | -9.157 | 0.3503 | -26.14 | 0.0000 | *** |
| $FG$ | -12.22 | 0.3603 | -33.91 | 0.0000 | *** |
| $DZ$ | -11.19 | 0.4018 | -27.85 | 0.0000 | *** |
| $RA$ | -9.436 | 0.3533 | -26.71 | 0.0000 | *** |
| $SN$ | -10.21 | 0.3709 | -27.52 | 0.0000 | *** |
| $Z_{{PM}_{2.5}.}$ | -0.9447 | 0.01896 | -49.83 | 0.0000 | *** |
| $Z_{CPM}$ | 1.502 | 0.7512 | 2 | 0.0455 | * |
| $Z_{TMP}:Z_{CPM}$ | -0.09854 | 0.02801 | -3.518 | 0.0004 | *** |
| $Z_{RH}:Z_{CPM}$ | 0.278 | 0.02474 | 11.24 | 0.0000 | *** |
| $Z_{WS}:Z_{CPM}$ | 0.01764 | 0.01098 | 1.605 | 0.1084 |  |
| $HZ:Z_{CPM}$ | -2.087 | 0.7537 | -2.769 | 0.0056 | ** |
| $DU:Z_{CPM}$ | -1.738 | 0.7527 | -2.31 | 0.0209 | * |
| $BR:Z_{CPM}$ | -1.982 | 0.7518 | -2.637 | 0.0084 | ** |
| $FG:Z_{CPM}$ | -1.257 | 0.7686 | -1.636 | 0.1019 |  |
| $DZ:Z_{CPM}$ | -1.798 | 0.8348 | -2.154 | 0.0312 | * |
| $RA:Z_{CPM}$ | -2.056 | 0.7574 | -2.715 | 0.0066 | ** |
| $SN:Z_{CPM}$ | -1.418 | 0.7853 | -1.806 | 0.0710 | . |

1. Model 6

|  | Estimate | Std. Error | z-value | p-value^a^ |  |
| --- | --- | --- | --- | --- | --- |
| (Intercept):1 | 17.71 | 0.3367 | 52.6 | 0.0000 | *** |
| (Intercept):2 | 0.5378 | 0.007092 | 75.82 | 0.0000 | *** |
| $Z_{TMP}$ | 0.03373 | 0.02599 | 1.298 | 0.1944 |  |
| $Z_{RH}$ | -2.879 | 0.04512 | -63.8 | 0.0000 | *** |
| $Z_{WS}$ | -0.05516 | 0.02042 | -2.701 | 0.0069 | ** |
| $HZ$ | -10.19 | 0.342 | -29.8 | 0.0000 | *** |
| $DU$ | -8.72 | 0.4127 | -21.13 | 0.0000 | *** |
| $BR$ | -8.87 | 0.3346 | -26.51 | 0.0000 | *** |
| $FG$ | -12.06 | 0.3463 | -34.82 | 0.0000 | *** |
| $DZ$ | -10.76 | 0.3896 | -27.61 | 0.0000 | *** |
| $RA$ | -9.222 | 0.3372 | -27.35 | 0.0000 | *** |
| $SN$ | -9.821 | 0.3552 | -27.65 | 0.0000 | *** |
| $Z_{{PM}_{2.5}.}$ | -1.448 | 0.192 | -7.543 | 0.0000 | *** |
| $Z_{CPM}$ | 1.495 | 0.7189 | 2.079 | 0.0376 | * |
| $Z_{TMP}:Z_{{PM}_{2.5}}$ | -0.231 | 0.02006 | -11.52 | 0.0000 | *** |
| $Z_{RH}:Z_{{PM}_{2.5}}$ | 0.6756 | 0.03096 | 21.82 | 0.0000 | *** |
| $Z_{WS}:Z_{{PM}_{2.5}}$ | 0.08626 | 0.01615 | 5.341 | 0.0000 | *** |
| $HZ:Z_{{PM}_{2.5}}$ | 0.6643 | 0.1958 | 3.393 | 0.0007 | *** |
| $DU:Z_{{PM}_{2.5}}$ | -0.005817 | 0.2274 | -0.026 | 0.9796 |  |
| $BR:Z_{{PM}_{2.5}}$ | -0.2082 | 0.1911 | -1.09 | 0.2759 |  |
| $FG:Z_{{PM}_{2.5}}$ | 0.3501 | 0.213 | 1.643 | 0.1003 |  |
| $DZ:Z_{{PM}_{2.5}}$ | -0.3431 | 0.2995 | -1.145 | 0.2520 |  |
| $RA:Z_{{PM}_{2.5}}$ | -0.5826 | 0.1985 | -2.935 | 0.0033 | ** |
| $SN:Z_{{PM}_{2.5}}$ | -0.2607 | 0.2279 | -1.144 | 0.2525 |  |
| $Z_{TMP}:Z_{CPM}$ | 0.02012 | 0.02888 | 0.697 | 0.4860 |  |
| $Z_{RH}:Z_{CPM}$ | 0.193 | 0.02512 | 7.684 | 0.0000 | *** |
| $Z_{WS}:Z_{CPM}$ | -0.02346 | 0.01112 | -2.109 | 0.0349 | * |
| $HZ:Z_{CPM}$ | -2.132 | 0.7217 | -2.954 | 0.0031 | ** |
| $DU:Z_{CPM}$ | -1.595 | 0.7204 | -2.213 | 0.0269 | * |
| $BR:Z_{CPM}$ | -1.863 | 0.7197 | -2.588 | 0.0097 | ** |
| $FG:Z_{CPM}$ | -1.972 | 0.7413 | -2.661 | 0.0078 | ** |
| $DZ:Z_{CPM}$ | -1.878 | 0.8274 | -2.269 | 0.0233 | * |
| $RA:Z_{CPM}$ | -1.726 | 0.7269 | -2.374 | 0.0176 | * |
| $SN:Z_{CPM}$ | -1.24 | 0.753 | -1.647 | 0.0995 | . |

Table S-7. Effect of a specific PM concentration on visibility under each weather condition (from Model 6)

|  |  | Weather | PM effect on visibility (km) | | | |
| --- | --- | --- | --- | --- | --- | --- |
| ICN | PM_2.5_ | HZ | –0.784 | $\mathbf{- 0.2310} \left[ Z_{TMP} \right]$ | $\boldsymbol{+ 0.6756} \left[ Z_{RH} \right]$ | $+ 0.08626 \left[ Z_{WS} \right]$ |
|  |  | DU | –1.454 | “ | “ | “ |
|  |  | BR | –1.656 | “ | “ | “ |
|  |  | FG | –1.098 | “ | “ | “ |
|  |  | DZ | –1.791 | “ | “ | “ |
|  |  | RA | –2.031 | “ | “ | “ |
|  |  | SN | –1.709 | “ | “ | “ |
|  | CPM | HZ | –0.637 | $+ 0.02012 \left[ Z_{TMP} \right]$ | $+ \boldsymbol{0.1930} \left[ Z_{RH} \right]$ | $- 0.02346 \left[ Z_{WS} \right]$ |
|  |  | DU | –0.100 | “ | “ | “ |
|  |  | BR | –0.368 | “ | “ | “ |
|  |  | FG | –0.477 | “ | “ | “ |
|  |  | DZ | –0.383 | “ | “ | “ |
|  |  | RA | –0.231 | “ | “ | “ |
|  |  | SN | 0.255 | “ | “ | “ |

Table S-8. Mean squared error of predicted visibility (km) for each model under different weather conditions at the ICN in 2019

| **Model**  **No.** | HZ | DU | BR | FG | DZ | RA | SN | All |
| --- | --- | --- | --- | --- | --- | --- | --- | --- |
| 0 | 4.09 | 2.44 | 4.19 | 1.17 | 6.04 | 6.71 | 7.65 | 1.47 |
| 1 | 1.99 | 1.55 | 2.21 | 1.49 | 5.13 | 5.30 | 5.65 | 0.91 |
| 2 | **1.94** | **1.27** | 2.14 | 1.06 | 4.65 | 4.55 | 4.91 | **0.83** |
| 3 | 2.01 | 2.35 | 2.21 | 1.50 | 5.10 | 5.26 | 5.65 | 0.92 |
| 4 | 1.97 | 1.91 | 2.14 | 1.05 | 4.64 | 4.52 | 4.93 | 0.84 |
| 5 | 2.18 | 5.66 | 2.20 | 1.52 | 4.93 | 5.04 | 5.44 | 0.93 |
| 6 | 2.28 | 2.45 | **2.13** | **1.04** | **4.51** | **4.47** | **4.84** | 0.86 |


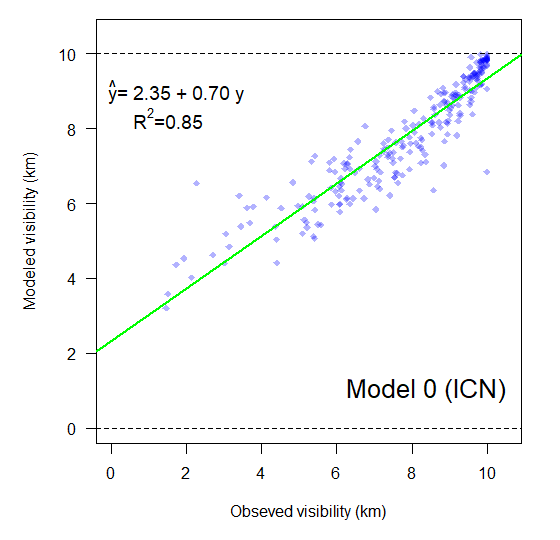


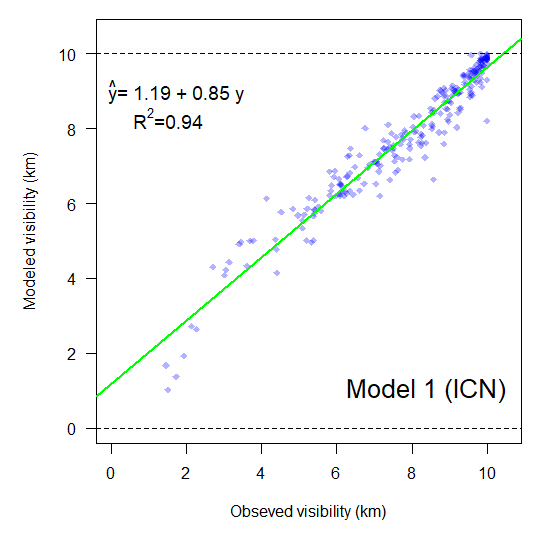

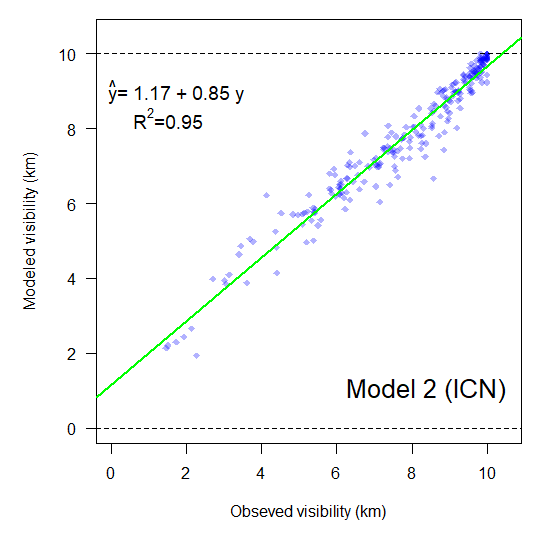


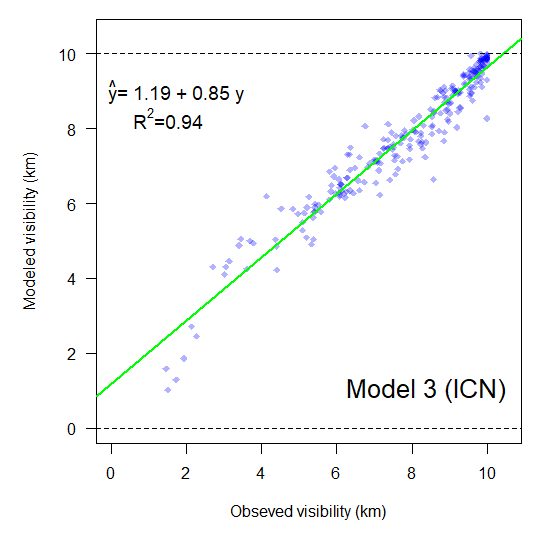

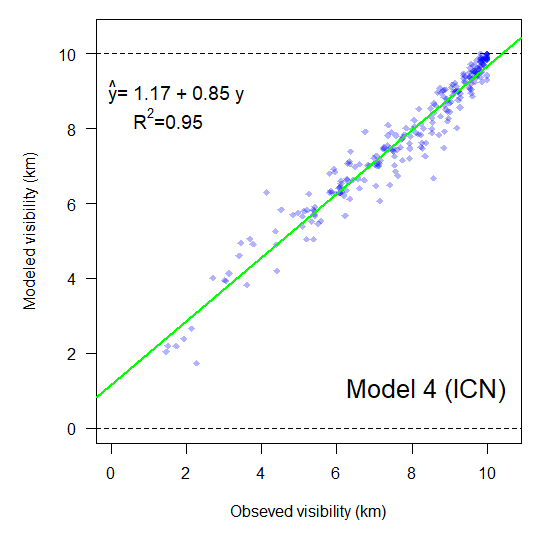


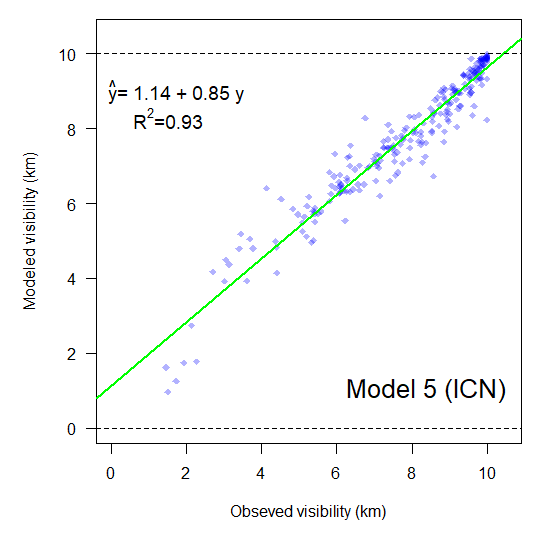

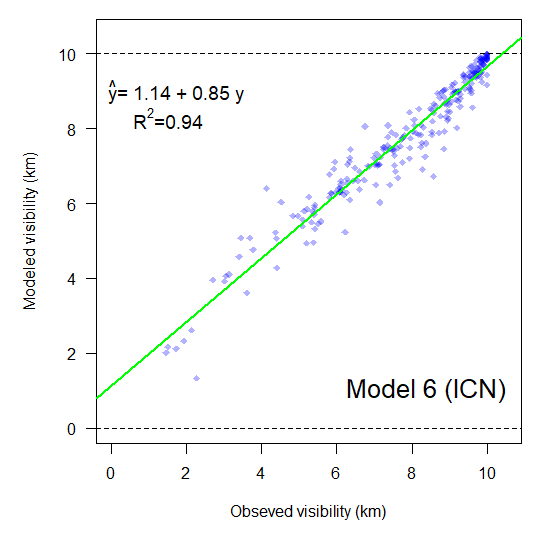


Fig. S-3. Daily averages of the modeled and observed hourly visibility at the ICN in 2019


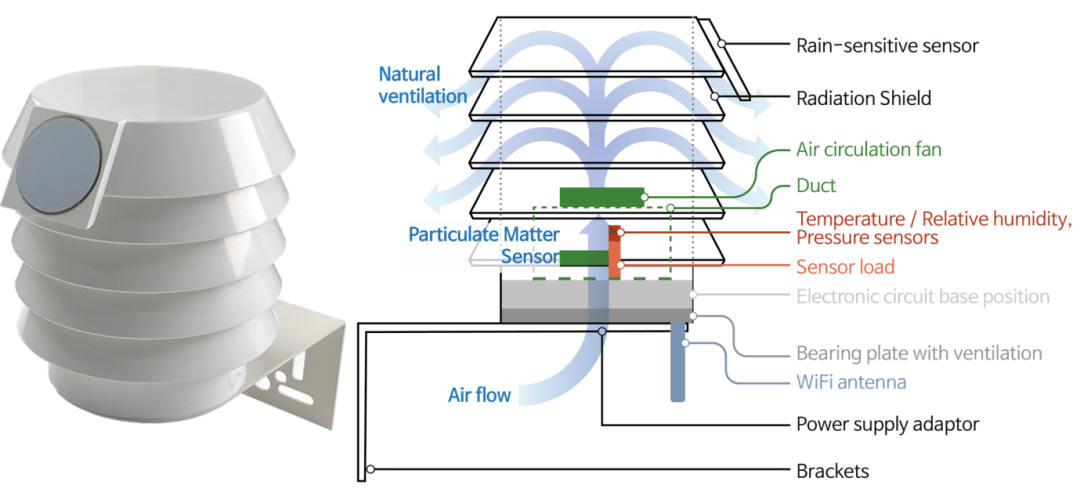


Fig. S-4. Low-cost sensor monitor, *‘W-station*’ (Observer Co.), used for field measurements.

Table S-9. Specifications of the PM_2.5_ sensor, *SPS30* (Sensirion)

| Condition | Value | |
| --- | --- | --- |
| Sensor Type | Laser-based light scattering particle sensing | |
| Mass range | 0–1,000 μg m^-3^ | |
| Accuracy | 0–100 μg m^-3^ | ±10 μg m^-3^ |
|  | 100–1,000 μg m^-3^ | ±10% |
| Size range | PM_2.5_ | 0.3 to 2.5 μm |
| Ventilation | Fan aspirated | |


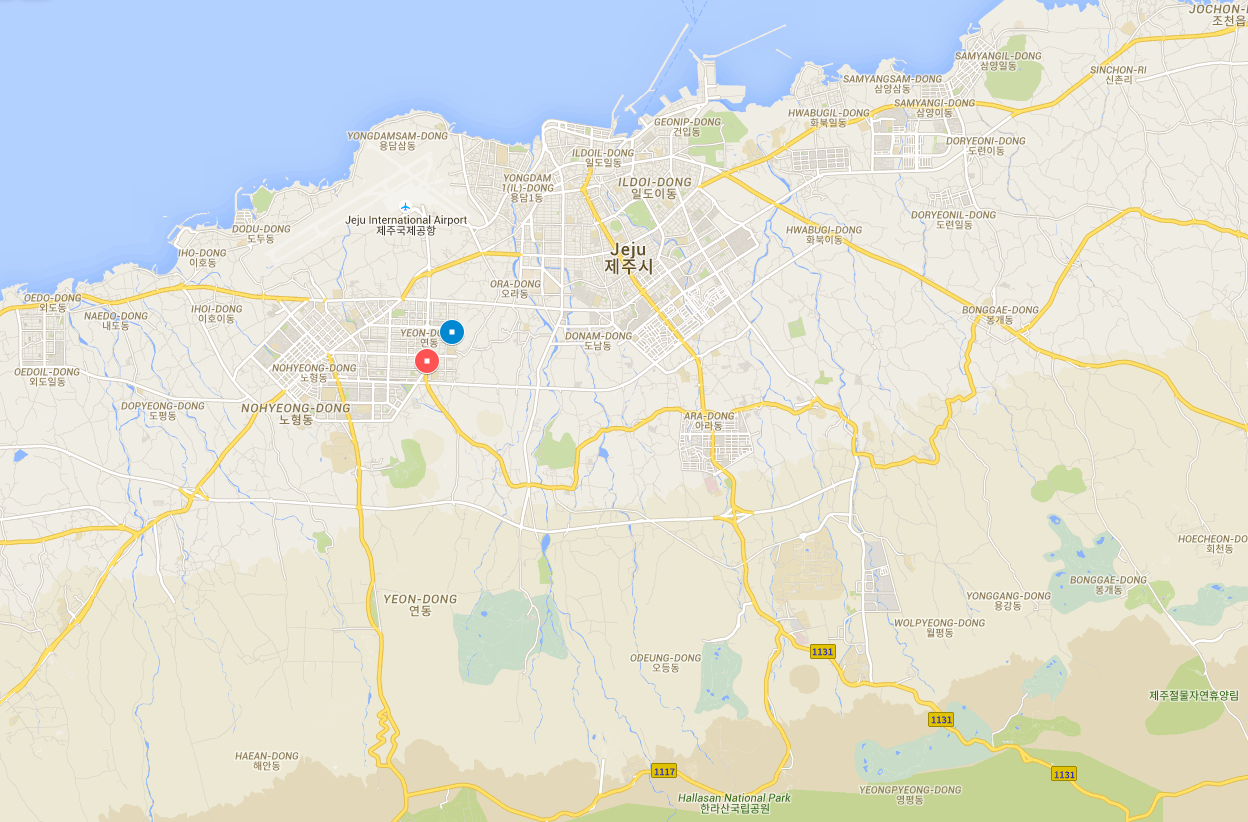


1. Jeju, Korea (March 25 – October 25, 2019); Map data ©2021 Google My Maps


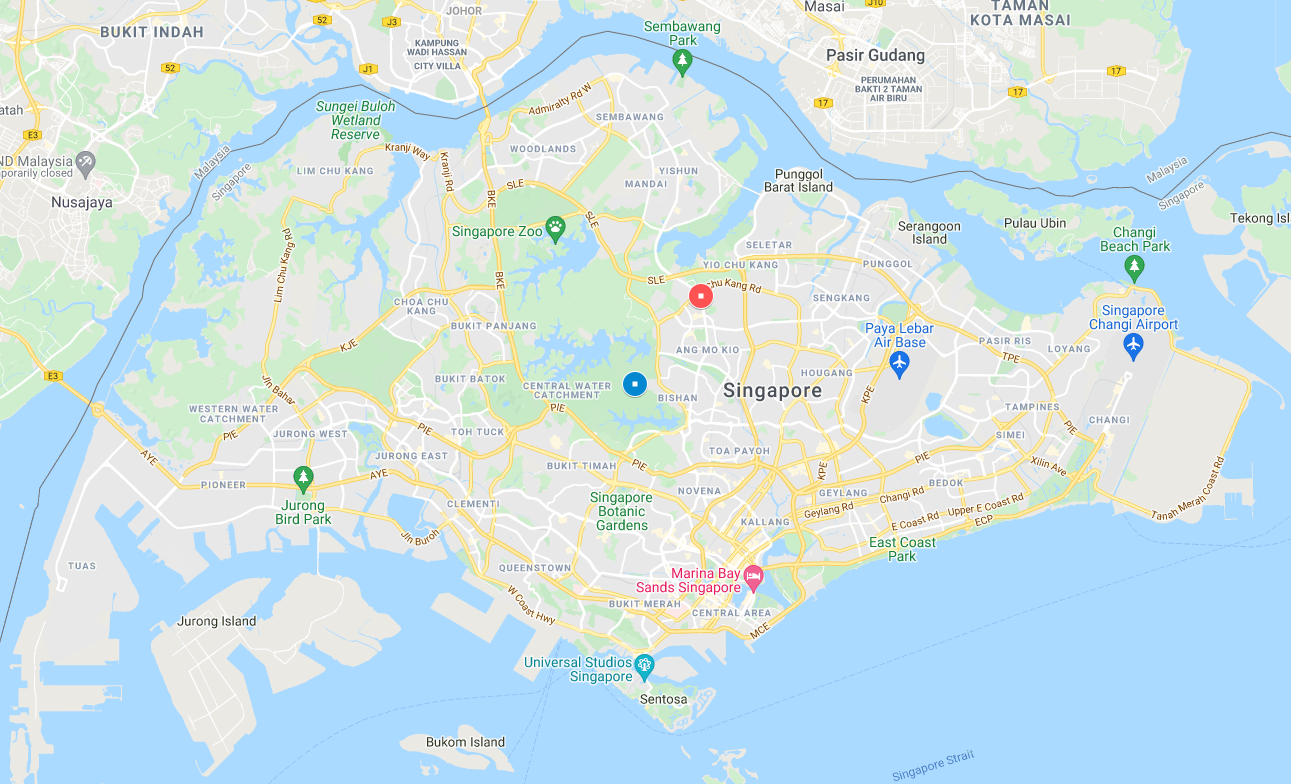


(b) Singapore (December 1 – March 31, 2021); Map data ©2021 Google My Maps

Fig. S-5. Field measurement study period and locations of the low-cost sensor (red circle) and reference air-quality monitoring station (blue circle) in (a) Jeju, Korea and (b) Singapore (Google, n.d.-a; b). The distances between the low-cost sensor and the reference station are 0.6 km and 4.2 km in Jeju and Singapore, respectively: 33.485°N, 126.496°E and 33.489°N, 126.501°E in Jeju; 1.388°N, 103.843°E and 1.357°N, 103.820°E in Singapore.

References

NGII. (2020). Geospatial Information Service Platform. Retrieved November 16, 2020, from http://map.ngii.go.kr/ms/map/NlipMap.do, National Geographic Information Institute, Korea

Google. (n.d.-a). Google Maps Jeju-si. Retrieved April 21, 2021, from https://goo.gl/maps/DGZLR2bJfP7A7hhw6

Google. (n.d.-b). Google Maps Singapore. Retrieved April 21, 2021, from https://goo.gl/maps/N5Ti7TieUJyu5jcW6
